# Polymeric Microarray Patches for Enhanced Transdermal Delivery of the Poorly Soluble Drug Olanzapine

**DOI:** 10.1021/acsami.3c05553

**Published:** 2023-06-22

**Authors:** Peter E. McKenna, Marco T. A. Abbate, Lalit K. Vora, Akmal H. Sabri, Ke Peng, Fabiana Volpe-Zanutto, Ismaiel A. Tekko, Andi Dian Permana, Cian Maguire, David Dineen, Mary-Carmel Kearney, Eneko Larrañeta, Alejandro J. Paredes, Ryan F. Donnelly

**Affiliations:** † School of Pharmacy, Queen’s University Belfast, Medical Biology Centre, 97 Lisburn Road, Belfast BT9 7BL, United Kingdom; ‡ Department of Pharmaceutics and Pharmaceutical Technology, Faculty of Pharmacy, Aleppo University, Aleppo 6458+5CM, Syria; § Department of Pharmaceutics, Faculty of Pharmacy, Hasanuddin University, Jalan Perintis Kemerdekaan KM 10, Tamalanrea Indah Kec., Kota Makassar, Sulawesi Selatan Makassar 90245, Indonesia

**Keywords:** Transdermal, Polymeric, Microneedle, Solubility, Cyclodextrin, Nanocrystal, Antipsychotic

## Abstract

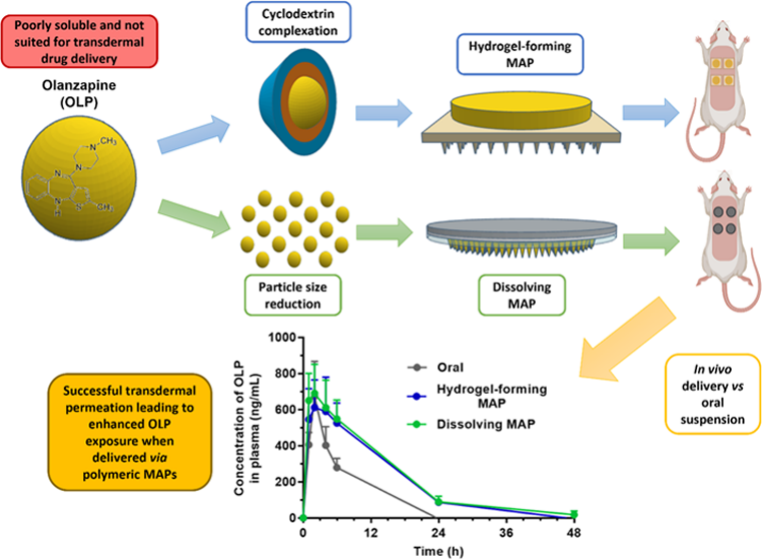

Transdermal drug delivery is an alternative route of administration that offers avoidance of the associated drawbacks of orally and parenterally administered hydrophobics. However, owing to the extremely specific set of physicochemical characteristics required for passive transdermal drug permeation, the development of marketed transdermal products containing poorly soluble drugs has been severely limited. Microarray patches (MAPs) are a type of transdermal patch that differ from the traditional patch design due to the presence of tiny, micron-sized needles that permit enhanced drug permeation on their application surface. To date, MAPs have predominantly been used to deliver hydrophilic compounds. However, this work challenges this trend and focuses on the use of MAPs, in combination with commonly utilized solubility-enhancing techniques, to deliver the hydrophobic drug olanzapine (OLP) across the skin. Specifically, cyclodextrin (CD) complexation and particle size reduction were employed in tandem with hydrogel-forming and dissolving MAPs, respectively. *In vivo* experimentation using a female Sprague-Dawley rat model confirmed the successful delivery of OLP from hydrogel-forming MAPs (*C*_max_ = 611.13 ± 153.34 ng/mL, *T*_max_ = 2 h) and dissolving MAPs (*C*_max_ = 690.56 ± 161.33 ng/mL, *T*_max_ = 2 h) in a manner similar to that of oral therapy in terms of the rate and extent of drug absorption, as well as overall drug exposure and bioavailability. This work is the first reported use of polymeric MAPs in combination with the solubility-enhancing techniques of CD complexation and particle size reduction to successfully deliver the poorly soluble drug OLP *via* the transdermal route. Accordingly, this paper provides significant evidence to support an expansion of the library of molecules amenable to MAP-mediated drug delivery to include those that exhibit poor aqueous solubility.

## 1 Introduction

Currently, poor drug solubility is one of the most common physicochemical obstacles faced by formulation scientists as they work toward bringing drug products to market with approximately 40–70% of new chemical entities and 90% of current drug candidates exhibiting poor aqueous solubility.^1,2^ Oral drug formulations possess many advantages, including convenient, non-invasive, and pain-free administration, cost-effective manufacture, ease of formulation, and suitability for industrial scale-up.^3^ However, despite the advantages of oral drug delivery, it is not bereft of shortcomings. Problems can arise from patient swallowing difficulties, drug degradation upon exposure to harsh environments of the GI tract, and changes in drug absorption depending on stomach contents and the rate of gastric emptying.^4,5^ Furthermore, the administration of poorly soluble drug molecules *via* the oral route is associated with reduced drug absorption and, therefore, bioavailability.^6^ Injectable therapies, namely, those that are administered intravenously, intramuscularly, intradermally, or subcutaneously, are commonly used when oral drug delivery is not a viable option.^7^ Considering the injection of poorly soluble compounds, significant advantages include the potential for direct administration to the system circulation (100% bioavailability) and the ease at which extended release preparations can be formulated.^8^ Nevertheless, much like oral formulations, there are multiple drawbacks associated with the use of injectable therapies, such as needle-phobia, sharps waste generation, and the risk of infectious disease transmission.^9,10^

Transdermal drug delivery is an alternative method of drug delivery that may bypass many of the drawbacks associated with oral and injectable therapies. Transdermal formulations are easily self-applied, typically non-/minimally invasive in nature, and generate no sharps waste following use.^10,11^ Moreover, unlike oral drug delivery, first-pass metabolism, drug degradation caused by exposure to the GI tract and the occurrence of GI side effects, such as nausea and diarrhea, is avoided.^12^ Nevertheless, due to the highly effective barrier properties of the outermost layer of the skin, known as the *stratum corneum*, the number of molecules suited to delivery across the skin in a passive manner is severely limited. As a result, transdermal permeation enhancement strategies represent one of the most intensely researched areas in the field of transdermal drug delivery. Microarray patches (MAPs) are an active strategy for the enhancement of transdermal permeation. These transdermal patch-type systems are composed of a flat baseplate from which an array of small needles, with heights in the micrometer range, protrude outward in a perpendicular fashion.^13,14^ Upon application to the skin, these MNs painlessly, and without drawing blood, puncture the *stratum corneum* to create temporary conduits in the skin’s primary barrier through which drug molecules can be delivered. Currently, there are five MAP types, namely, solid, coated, hollow, hydrogel-forming, and dissolving.^10,15^ This work was focused on the two latter MAP types, *i.e.*, hydrogel-forming and dissolving. Hydrogel-forming MAPs consist of a highly swellable MN array composed of cross-linked polymers, atop which is fixed a separately formulated drug reservoir.^16^ Upon application to the skin, MNs rapidly imbibe interstitial fluid and swell to form an aqueous hydrogel matrix *in situ*. When the MN array is sufficiently swollen, the affixed drug reservoir dissolves, resulting in drug diffusion into and through the hydrogel matrix before subsequent delivery into the viable epidermis. Dissolving MAPs, as their name suggests, are composed of a soluble/biodegradable matrix that, upon application to the skin, breaks down to release their incorporated drug cargo intradermally.

In this work, the antipsychotic drug olanzapine (OLP) was used as a model compound to demonstrate how the use of dissolving and hydrogel-forming MAPs, in combination with commonly utilized solubility enhancement strategies, can facilitate extensive transdermal delivery of a poorly soluble drug. In the case of hydrogel-forming MAPs, solubility enhancement of OLP through complexation with hydroxypropyl-β-cyclodextrin (HP-β-CD) was achieved using multiple techniques before OLP/HP-β-CD complex-containing directly compressed tablets (DCTs) were manufactured and subsequently combined with hydrogel-forming MN arrays. Following this, and separately, particle size reduction of OLP using a top-down wet bead milling approach was employed to fabricate drug nanocrystals (NCs) with enhanced aqueous solubility that were then formulated into robust dissolving MAPs. Both MAP types were characterized *in vitro* after which the delivery of OLP from each was assessed *in vivo* using a Sprague-Dawley rat model. The aim of this work was to highlight the adaptive potential of polymeric MAPs as an alternative platform for the delivery of molecules that demonstrate poor aqueous solubility.

## 2 Materials and Methods

### 2.1 Materials

The following materials were used in the study: OLP (Tokyo Chemical Industry, Zwijndrecht, Belgium); Gantrez S-97 (a copolymer of methyl vinyl ether and maleic acid), PVP (58 and 360 kDa, sold under the product brand names Plasdone K-29/32 and K-90), and β-CD, HP-β-CD, γ-CD, and HP-γ-CD (sold under the product brand names of Cavamax W7, Cavitron W7, Cavamax W8, and Cavasol W8, respectively, Ashland, Vale Industrial Estate, Kidderminster, U.K.); sorbitol, poly(ethylene glycol) (PEG, MW 3400 and 10,000 Da), poly(vinyl alcohol) (PVA, MW 85–124 kDa, 87–89% hydrolyzed), PVA (MW 9–10 kDa, 80% hydrolyzed), and d-α-tocopherol poly(ethylene glycol) 1000 succinate (TPGS, Sigma-Aldrich, Steinheim, Germany); crospovidone (CPV, sold under the product brand name Kollidon CL-SF, O-BASF, Ludwigshafen, Germany); anhydrous citric acid, anhydrous glucose, and anhydrous sodium carbonate (Na_2_CO_3_) (BDH Laboratory Supplies, Poole, Dorset, England); Parafilm M laboratory film (Bemis Company Inc., Soignies, Belgium); siliconized release liner (Rexam Release B.V., Apeldoom, The Netherlands); pluronic F-108 (VWR Chemicals LLC, Solon, OH); poly(lactic acid) (PLA) (Ultimaker, Geldermalsen, Netherlands); Kinesiology Sports tape (95% cotton, 5% spandex; 5 × 500 cm; Proworks Corporation, Corvallis, OR); and microfoam surgical tape and Tegaderm dressing (3 M, Bracknell, Berkshire, U.K.).

### 2.2 Hydrogel-Forming MAPs

As stated previously, a hydrogel-forming MAP is composed of an MN array and a separately formulated drug-containing reservoir. In this work, the performance of two different hydrogel-forming MN array types with a variety of DCT drug reservoirs was assessed in an *in vitro* setting, with the most promising combination taken forward to *in vivo* investigation.

#### 2.2.1 Preparation of Hydrogel-Forming MN Arrays

**Figure 1:**
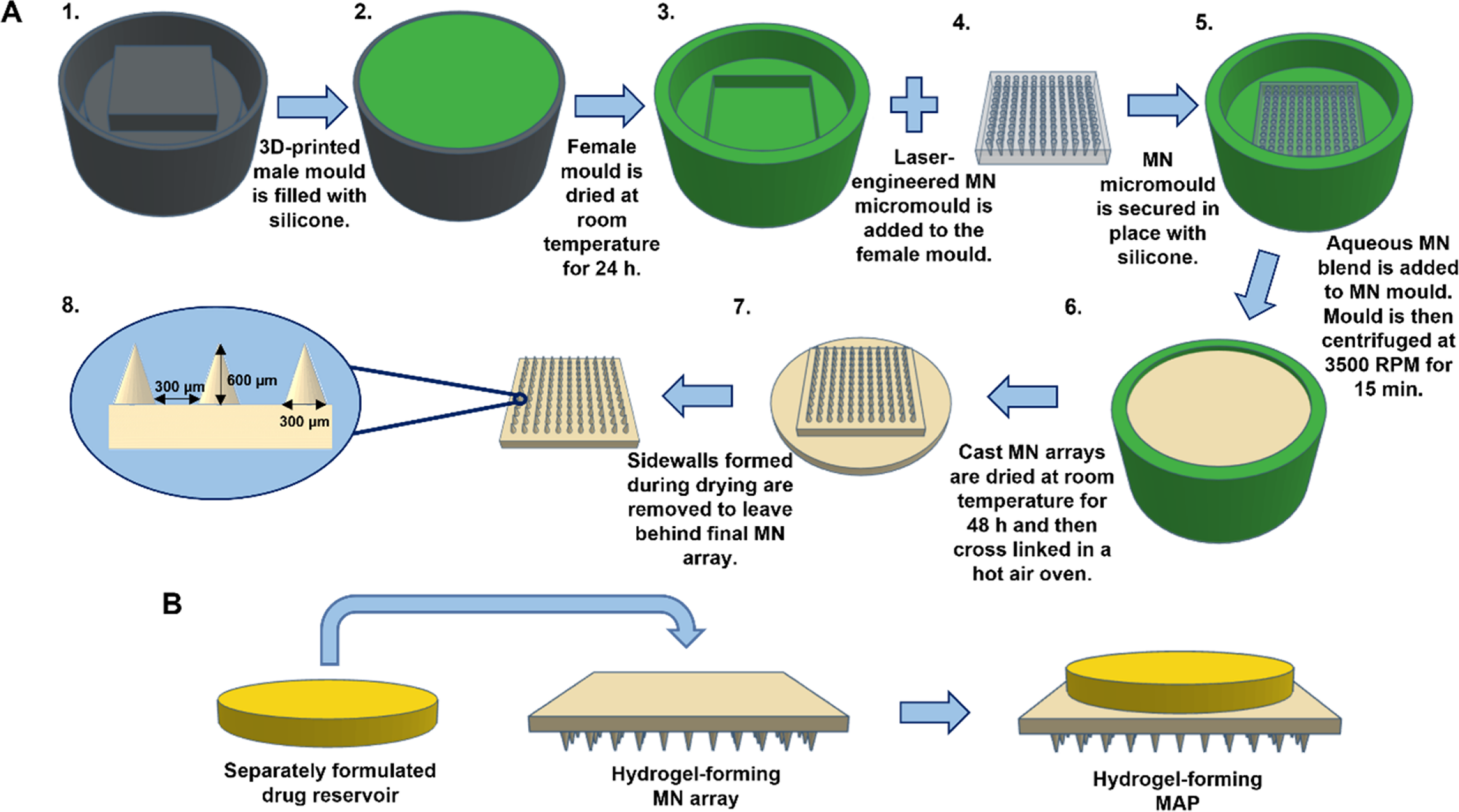
Schematic representation of (A) hydrogel-forming microneedle array fabrication and (B) assembly of a separately formulated drug reservoir and a hydrogel-forming microneedle array to form a hydrogel-forming microarray patch.

Hydrogel-forming MN arrays were prepared by the casting of aqueous polymer blends into laser-engineered micromoulds, where they were dried and then subsequently cross-linked according to the conditions detailed in Table 1. The first formulation was cast from an aqueous blend of Gantrez S-97 (20% w/w), PEG 10,000 (7.5% w/w), and the pore-forming agent anhydrous sodium carbonate (Na_2_CO_3_, 3% w/w).^16^ The second formulation was cast from an aqueous blend of 15% w/w PVA (MW 85–124 kDa, 87–89% hydrolyzed), 10% w/w PVP (MW 58 kDa), and anhydrous citric acid (1.5% w/w).^17^ The final arrays produced (Figure 1) were composed of 121 needles arranged in an 11 × 11 formation, perpendicular to the base and of conical shape (∼600 μm in height, with base width of 300 μm, and 300 μm interspacing on a 0.49 cm^2^ array).

**Table 1:** Cross-Linking Conditions for Each of the Hydrogel Formulations Tested

formulation	oven temperature (°C)	time (h)
Gantrez	80	24
PVA/PVP	130	3

#### 2.2.2 Characterization of Hydrogel-Forming MN Arrays

##### 2.2.2.1 Visual Inspection

Following fabrication, and prior to use in both *in vitro* and *in vivo* studies, fabricated MN arrays were viewed under a digital light microscope (Leica Microsystems, Milton Keynes, Buckinghamshire, U.K.) with the height of individual MNs measured and recorded as *H*_b_.

##### 2.2.2.2 Insertion Profile and MN Height Reduction

To test the insertion efficiency of the formulated MN arrays, a TA.XT.Plus Texture Analyser (Stable MicroSystems Ltd., Godalming, Surrey, U.K.) and an artificial skin model composed of eight layers of Parafilm M were used according to the widely accepted protocol previously reported by Larrañeta et al.^18^ Insertion efficiency was calculated using eq 1
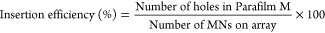
1Additionally, MN height reduction after insertion into the artificial Parafilm M skin model was carried out by visualizing MNs post-insertion using a Leica EZ4 D digital microscope, measuring individual MN heights (*H*_a_), and comparing these with MN heights before insertion (*H*_b_) using eq 2
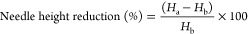
2The insertion depth of MNs into full-thickness neonatal porcine skin was assessed using the same insertion protocol as previously. An OCT microscope was then used to obtain cross-sectional images of the inserted MNs, and ImageJ software (National Institutes of Health, Bethesda, MD) was used to process the obtained images.

##### 2.2.2.3 Swelling Profile

Determination of the swelling profile of each hydrogel formulation was conducted using an excess volume of phosphate-buffered saline (PBS) (pH 7.4), as described previously by Donnelly et al., (2014).^16^ The percentage swelling of each film was calculated using eq 3, where *M*_0_ represents the starting mass of the hydrogel film and *M*_t_ represents the mass of the swollen hydrogel film at a given timepoint
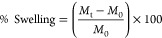
3

#### 2.2.3 Preparation of Separately Formulated Drug Reservoirs

Multiple different reservoir types, namely, polymeric films,^19^ lyophilized wafers,^16^ solid-dispersion tablets,^20^ and liquid reservoirs,^21^ have been successfully used in combination with hydrogel-forming MAPs. In this work, direct compression was selected as a low-cost, efficient, and scalable method of forming solid DCT reservoirs containing OLP (either alone or complexed with HP-β-CD) and commonly used pharmaceutical excipients.

##### 2.2.3.1 OLP/CD Phase-Solubility Analysis

Cyclodextrin (CD) complexation was the strategy chosen to enhance the solubility of OLP when loaded into DCT drug reservoirs. To investigate the ability of various types of CD to complex with and, therefore, enhance the solubility of OLP, phase-solubility studies were carried out according to the methodology developed by Higuchi and Connors.^22^ Briefly, excess amounts of OLP (∼30 mg) were added to 5 mL aliquots of PBS (pH 7.4) containing successively increasing concentrations (0, 5, 10, 15, 20, 25, and 50 mMol/L) of β-CD, HP-β-CD, γ-CD, or HP-γ-CD in snap-top glass vials. Vials were mixed by vortexing for 1 min and then placed in an oscillating incubator (GFL, Burgwedel, Germany) for 24 h (37 ± 1 °C and 40 RPM). Following incubation, the samples were removed, filtered through a 0.2 μm poly(tetrafluoroethylene) (PTFE) syringe filter, and diluted appropriately before analysis using ultraviolet-high-performance liquid chromatography (UV-HPLC).

##### 2.2.3.2 Formation of OLP/CD Inclusion Complexes in the Solid State

Following phase-solubility investigations, exploration into the formation of OLP/CD complexes in the solid state, *i.e.*, in a powdered form, was carried out. Importantly, this work involved the use of organic solvents in which both OLP and HP-β-CD were highly soluble. It was hypothesized that, due to an increase in the amount of OLP in solution, a greater level of successful inclusion complex formation would be achieved upon solvent removal. This study involved the exposure of a physical mixture (PM) containing OLP and HP-β-CD in a 1:1 molar ratio to the previously reported complexation processes of kneading (KN), freeze-drying (FD) and coevaporation (COEV). Additionally, organic solvent coevaporation (M-COEV) and spray-drying (SD), two previously unreported methods of complexation, were tested in the same manner. In all cases, harvested OLP/HP-β-CD complexes were stored away from light and in airtight containers until further use.

##### 2.2.3.2.1 Kneading Method (KN)

An OLP/HP-β-CD PM, prepared as described previously, was placed in a mortar and dissolved using the minimum required volume of ethanol/water (50% v/v). This wetted mixture was then kneaded with a pestle for 15 min until a dense yellow paste was formed, which was then dried in a hot air oven at 80 °C for 2 h.^23^

##### 2.2.3.2.2 Coevaporation Method (COEV)

The required amount of OLP was dissolved in a minimum volume of methanol. This OLP solution was then added in a dropwise manner to an aqueous solution of HP-β-CD dissolved in a minimum volume of water. The resultant solution was mixed by repeated inversion for 1 h. Finally, both solvents were eliminated under vacuum in a BUSHI Rotavapor R-200 rotary evaporator (BUSHI Ltd., Postfach, Switzerland) at 40 °C.^23^

##### 2.2.3.2.3 Freeze-Drying Method (FD)

FD samples were prepared in a similar manner to COEV samples. Prior to freeze-drying, methanol was eliminated under vacuum in a rotary evaporator at 40 °C. Following organic solvent elimination, 5 mL aliquots of the residual aqueous solution were frozen at −80 °C for 60 min and then lyophilized in a Virtis Advantage Bench Top Freeze Drier (SP Scientific Ltd., Warminster, PA). The following freeze-drying regime was implemented: primary drying at −40 °C for 90 min, followed by 90 min at −30 °C, then for 90 min at −20 °C, next for 530 min at −10 °C, and finally for 90 min at 0–10 °C. Secondary drying for 660 min at a shelf temperature of 25 °C was then carried out. A vacuum pressure of 50 mTorr was maintained throughout the process.^16^

##### 2.2.3.2.4 Organic Solvent Coevaporation Method (M-COEV)

A PM of OLP and HP-β-CD was dissolved in a minimum volume of methanol and then mixed by repeated inversion for 1 h. This OLP/HP-β-CD solution was then placed in a round-bottom flask and methanol was removed under vacuum in a BUSHI Rotavapor R-200 rotary evaporator at 40 °C. The dried OLP/HP-β-CD inclusion complex was then harvested from the surface of the round-bottom flask by scraping with a spatula.

##### 2.2.3.2.5 Organic Solvent Spray-Drying Method (SD)

SD samples were prepared according to the protocol outlined for M-COEV samples. However, instead of removing methanol *via* coevaporation, samples were spray-dried using the BÜSHI Nano Spray Dryer B-90 HP (BÜCHI Labortechnik AG., Meierseggstr. 40, Postfach CH-9230, Flawil, Switzerland) in closed loop mode due to the presence of the organic solvent. Spraying parameters were as follows: inlet temperature 66–68 °C, outlet temperature 38–40 °C, inert gas flow 80–150 L/min, internal pressure 30–60 hPa, and O_2_ concentration ∼ 1%.

##### 2.2.3.3 Characterization of OLP/CD Inclusion Complexes

Following complexation, the resultant product was analyzed in terms of its physicochemical properties to confirm the presence, or indeed absence, of amorphous content. Verification of an amorphous product was considered an indication of successful OLP/HP-β-CD inclusion complex formation.^24,25^

##### 2.2.3.3.1 Differential Scanning Calorimetry (DSC)

DSC analyses were performed on samples of pure OLP, pure HP-β-CD, OLP/HP-β-CD PM, and OLP/HP-β-CD inclusion complexes formed by KN, FD, COEV, M-COEV, and SD using an Advantage Model Q100 DSC (TA Instruments, New Castle, DE). Samples of 3–10 mg were weighed accurately and placed in aluminum pans that were then sealed by crimping and subsequently heated at a rate of 10 °C per min from 30 to 250 °C under a nitrogen flow of 50 mL/min.

##### 2.2.3.3.2 Powder X-ray Diffraction (PXRD)

PXRD analyses of pure OLP, pure HP-β-CD, OLP/HP-β-CD PM, and OLP/HP-β-CD inclusion complexes, formed by M-COEV and SD, were carried out using a MiniFlex II powder X-ray diffractometer with the PDWL software (Rigaku Corporation, Tokyo, Japan). Patterns were collected in continuous mode in the angular range of 3–45° 2θ, with a step size of 0.01°, a scanning rate of 2°/min, a voltage of 30 kV, and a current of 15 mA.

##### 2.2.3.3.3 Attenuated Total Reflection Fourier Transform Infrared (FTIR)

FTIR spectroscopy was used to analyze interactions present in pure OLP, pure CD, OLP/CD physical mixture, and OLP/HP-β-CD inclusion complexes formed by M-COEV and SD using an Accutrac FT/IR-4100 Series (Jasco, Essex, U.K.) equipped with MIRacle diamond ATR accessory (Pike Technologies Ltd., Madison, WI). The IR spectra were scanned and recorded in the region ranging from 4000 to 600 cm^–1^ at room temperature. Resolution was maintained at 4.0 cm^–1^ throughout the analysis and the obtained spectra were the result of an average of 64 scans.

##### 2.2.3.3.4 Scanning Electron Microscopy (SEM)

SEM images of pure OLP, pure CD, OLP/CD physical mixture, and OLP/HP-β-CD inclusion complexes formed by M-COEV, and SD were obtained under low vacuum at an excitation voltage of 15 kV and a 1000× magnification using a Hitachi TM3030 tabletop SEM microscope (Chiyoda-ku, Tokyo, Japan).

##### 2.2.3.3.5 Percentage Yield of Complexation Processes

The percentage yields of solid-state inclusion complexes obtained from the processes of M-COEV and SD were calculated using eq 4. In this equation, the solid mass before complexation is represented by *m*_a_, and the solid mass after complexation is represented by *m*_b_. This was carried out to assess the efficiency of each process in terms of the mass of powdered product obtained relative to the mass of starting reactants. Moreover, given the challenges associated with the translation of a novel formulation strategy from a research and development setting to a larger, industry-scale production lab, this was considered to be an indication of the suitability of each method for upscaling if this were to be required in the future
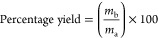
4

##### 2.2.3.3.6 Solubility Enhancement Inferred by CD Complexation

To investigate the solubility enhancement attributed to the formation of OLP/HP-β-CD inclusion complexes in the solid state, a study similar to the previously described phase-solubility analysis was carried out on complexes formed by SD. Specifically, the required mass of the solid-state OLP/HP-ß-CD inclusion complex, *i.e.*, the mass that ensured a concentration of HP-β-CD post-dissolution of 50 mMol/L, was accurately weighed and added to a snap-top glass vial. As before, 5 mL of PBS was then added to this snap-top glass vial and the sample was mixed by vortexing for 1 min, after which it was placed in an oscillating incubator (37 ± 1 °C and 40 RPM) for 24 h. Samples were taken at *t* = 1, 15, 30, 60, 180, and 1440 min, diluted appropriately, and analyzed using UV-HPLC (as detailed in Section 2.5).

#### 2.2.4 Characterization of Formulated DCTs

The composition of each of the DCT formulations tested during *in vitro* permeation studies is outlined in Table 2. Formulation FS represented a standard DCT formulation composed of OLP and anhydrous glucose. Anhydrous α-d-glucose is structurally similar to the α-d-glucopyranose units that make up HP-β-CD, but it does not possess any solubility-enhancing properties.^26^ In the formulation FCD, anhydrous glucose was replaced with HP-β-CD to investigate the effect of the addition of CD on the performance of DCTs. The effect of excipient use during DCT formulation was investigated by adding both sorbitol and crospovidone (CPV) to formulation FX and comparing the permeation of OLP from these DCTs with that of FCD and FS DCTs. The effect of forming solid-state OLP/HP-β-CD inclusion complexes *via* the novel spray-drying process outlined in Section 2.2.3.2.5 was investigated by comparing the permeation of OLP from SD DCTs to that from FX DCTs. Finally, PEG.SD DCTs were examined to deduce whether the inclusion of the water-soluble polymer PEG 3400 would improve the stability of the preformed inclusion complexes and, therefore, enhance OLP transit across the skin *via* a swollen hydrogel MAP. All DCTs were formed by the compression of 100 mg of the appropriate powder formulation in a manual hydraulic press at a force of 0.5 T for 20 s.

**Table 2:** Formulation Compositions of Directly Compressed Tablets Tested during *In Vitro* Permeation Studies

formulation	OLP (% w/w)	HP-β-CD (% w/w)	sorbitol (% w/w)	CPV (% w/w)	anhydrous glucose (% w/w)	PEG 3400 (% w/w)
FS	0.7	-	-	-	99.3	-
FCD	0.7	99.3	-	-	-	-
FX	0.7	78.3	7.0	14.0	-	-
SD	a0.7	a78.3	7.0	14.0	-	-
PEG.SD	a0.7	a78.3	-	-	-	a21.0

a Spray-dried according to the protocol outlined in Section 2.2.3.2.5.

##### 2.2.4.1 Dissolution Time and Drug Content

As an indication of the ability of each DCT formulation to dissolve or disintegrate in the case of FX DCTs, when in contact with a swollen hydrogel-forming MN array, the dissolution time of DCTs in PBS (pH 7.4) was assessed. Specifically, individual DCTs were placed in 20 mL of PBS (pH 7.4) with continuous stirring at 600 rpm and temperature maintained at 37 ± 1 °C. The time taken for each DCT to fully dissolve/disintegrate was recorded, after which OLP concentration was analyzed and DCT drug content was calculated.

##### 2.2.4.2 Hardness

DCT hardness was investigated using the ERWEKA TBH 125 tablet hardness tester (ERWEKA GmBH, Pittlerstraße, Germany). Briefly, individual DCTs were positioned on the stage of the tablet hardness tester, between two stainless steel probes, before a gradually incremental compression force was applied to the DCT by the probes. Upon DCT fracture, compression was ceased, and the fracture force automatically recorded. This was considered a practical assessment of DCT durability with regard to *in vitro* and *in vivo* experimentation as well as an indication of the viability of formulated DCTs throughout the processes of MAP assembly, transit, and application by a patient.

##### 2.2.4.3 Uniformity of Mass

Uniformity of mass of the formulated DCTs was investigated following the uniformity of mass for single-dose preparations test protocol as stated in the British Pharmacopoeia (BP).^27^ In accordance with this protocol, 20 DCTs from each formulation were selected randomly and weighed individually (*m*_a_). The mean mass was then calculated (*m*_b_) and, subsequently, the percentage mass deviation of each DCT from this mean mass was determined using eq 5
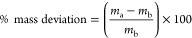
5As the mean DCT mass was between 80 and 250 mg, the acceptance criterion was such that when weighed individually, no more than two DCTs should deviate from the mean mass by more than 7.5%.^27^ Mean DCT dimensions, *i.e.*, thickness and diameter of the same randomly selected DCTs (*n* = 20), were measured using 0–150 mm digital callipers (Jade Products Rugby Ltd., Warwickshire, U.K.).

#### 2.2.5 *In Vitro* Delivery of OLP from Hydrogel-Forming MAPs

The Franz cell apparatus (PermeGear Inc., Sommerville, NJ) was used according to previously reported protocols to investigate the permeation of OLP from the five previously described DCT formulations *via* two types of hydrogel-forming MN arrays (Gantrez and PVA/PVP) across ethically obtained dermatomed neonatal porcine skin (Agri-Food and Biosciences Institute, Hillsborough, Ireland).^28^ To ensure that permeation studies were carried out under sink conditions, Franz cell apparatus receiver compartments were filled with prewarmed and degassed PBS (pH 7.4) containing HP-β-CD at a concentration of 5% w/v (37 ± 1°C). At predetermined intervals, 200 μL of the receiver medium was sampled from the Franz cell receiver compartment for analysis and replaced with 200 μL of fresh, prewarmed, and degassed receiver medium.

### 2.3 Dissolving MAPs

In this work, two types of dissolving MAPs were prepared, each with similar polymeric composition but containing OLP in different forms. The first dissolving MAP formulation, which was used as a control, contained unprocessed OLP, *i.e.*, crystalline OLP with varied particle sizes typically within the micrometer range, whereas the second formulation contained OLP NCs, *i.e.*, crystalline OLP with particle sizes in the nanometer range.

#### 2.3.1 Unprocessed OLP Particle Size Determination

The particle size of unprocessed OLP powder was determined by laser diffraction using a Malvern Mastersizer 3000 (Malvern Panalytical Ltd., Swords, Dublin, Ireland) with the corresponding Hydro-EV wet diffusion attachment as reported previously.^29^ Prior to analysis, a homogeneous suspension composed of 20 mg of OLP dispersed in 10 mL of an aqueous solution of Tween 80 (2% w/w) was prepared. An adequate volume of this suspension was then added to the beaker of deionized water until a laser obscuration value of 1.88% was obtained. Particle refractive index and particle absorption index were set at 1.709 and 1.000, respectively.

#### 2.3.2 Preparation of OLP Nanosuspension

The preparation of OLP NCs was achieved using wet bead milling in the presence of an aqueous surfactant solution (top-down methodology). The product of this process was an aqueous suspension containing OLP NCs, henceforth referred to as OLP nanosuspension (NS). Briefly, 5.5 g of zirconium beads, with diameter 0.1–0.2 mm, were added to a 7 mL snap-top glass vial. To this, 200 mg of unprocessed OLP powder was added followed by 5 mL of an aqueous surfactant solution. At this point, two magnetic sir-bars with dimensions 25 mm × 8 mm × 8 mm were placed inside the glass vial in an “X” formation. Finally, the glass vial containing all milling components was secured in place atop an IKA magnetic stirrer where milling was carried out at 1250 RPM for 24 h under ambient laboratory conditions. Following mill completion, the content of the glass vial was filtered through a nylon mesh to separate the zirconium beads from OLP NS. The beads, which were retained in the mesh, were then washed with 3 mL of deionized water to ensure greater yield of OLP NS. To determine the particle size and polydispersity index (PDI) of the formed OLP NCs, a Brookhaven Nano Omni particle size analyser (Brookhaven, New York, NY), which utilized dynamic light scattering (DLS), was used. Prior to analysis, a 4 μL aliquot of the filtered NS was dispersed in 3 mL of deionized water inside a plastic cuvette with analyses carried out under ambient conditions.

##### 2.3.2.1 Rationalization of Surfactant Selection

Selection of a surfactant that ensured the formation of OLP NCs with suitable size and stability was made based on the performance of three different candidates, *i.e.*, Pluronic F-108, d-α-tocopherol poly(ethylene glycol) 1000 succinate (TPGS), and a PVA/PVP (PVA 9–10 kDa, PVP 58 kDa) mixture. Aqueous solutions of each candidate surfactant were prepared at a concentration of 2% w/w and 5 mL of each was added to separate NS milling set-ups as detailed in Section 2.3.2. Milling and collection of each NS were carried out as detailed previously, with particle size and PDI obtained using the Brookhaven Nano Omni particle size analyser at *t* = 0, 2, 6, 24, 48, 72, 120, and 168 h. After 168 h, NS formulations that exhibited instability were discarded. Investigation of NC stability, in terms of particle size and PDI, was then extended to 3 months for the most stable NS formulation with measurements made at 14, 21, 28, 56, and 84 days.

##### 2.3.2.2 Freeze-Drying of OLP NS to Produce the OLP NC Powder

To remove water from the formulated OLP NS, thereby producing concentrated NC powder capable of redispersion and, therefore, formulation into a MAP, lyophilization was employed. Samples composed of OLP NS alone and samples composed of OLP NS with additional PVA/PVP 2% w/w solution were prepared in glass vials as detailed in Table 3.

**Table 3:** Composition of Olanzapine Nanosuspension Samples Processed *via* Freeze-Drying

formulation	volume of OLP NS (mL)	volume of PVA/PVP 2% w/w solution (mL)
NS1	1	0
NS2	1	0.25
NS3	1	0.5
NS4	1	0.75
NS5	1	1

The addition of PVA/PVP 2% w/w solution at this point facilitated investigation of the effect of surfactant concentration on the stability of OLP NCs during the freeze-drying process. Volumes of additional PVA/PVP 2% w/w solution in excess of 1 mL were not investigated as it was deemed that they may significantly reduce drug loading in the final dissolving MAP. Samples were frozen at −80 °C for 1 h before lyophilization according to the same protocol as detailed in Section 2.2.3.2.3. The particle size and PDI of OLP NCs after freeze-drying were determined and compared to those of OLP NCs that had not been freeze-dried, *i.e.*, OLP NS, to determine if the process of freeze-drying led to particle aggregation and, therefore, stability issues.

#### 2.3.3 Characterization of OLP NCs and Unprocessed OLP

In a similar manner to the characterization of drug-containing DCTs, each constituent of the formulated dissolving MAPs, *i.e.*, unprocessed OLP, PVA, PVP, a PM of these three components and OLP NC powder, were analyzed using DSC, PXRD, and SEM according to the protocols stated previously. Additionally, to determine the saturation solubility of OLP NCs, 100 mg of OLP NC powder (which contained ∼30 mg of OLP) was added to 5 mL of PBS (pH 7.4) and mixed by vortexing for 1 min. Samples were then placed in an oscillating incubator at 37 ± 1 °C and 40 RPM for 24 h. Samples were then removed, centrifuged at 5000 RPM for 10 min, and the supernatant filtered through a 0.2 μm PTFE syringe filter before appropriate dilution with ACN and analysis using HPLC-UV.

#### 2.3.4 Preparation of Dissolving MAPs Containing OLP NCs and Unprocessed OLP

**Figure 2:**
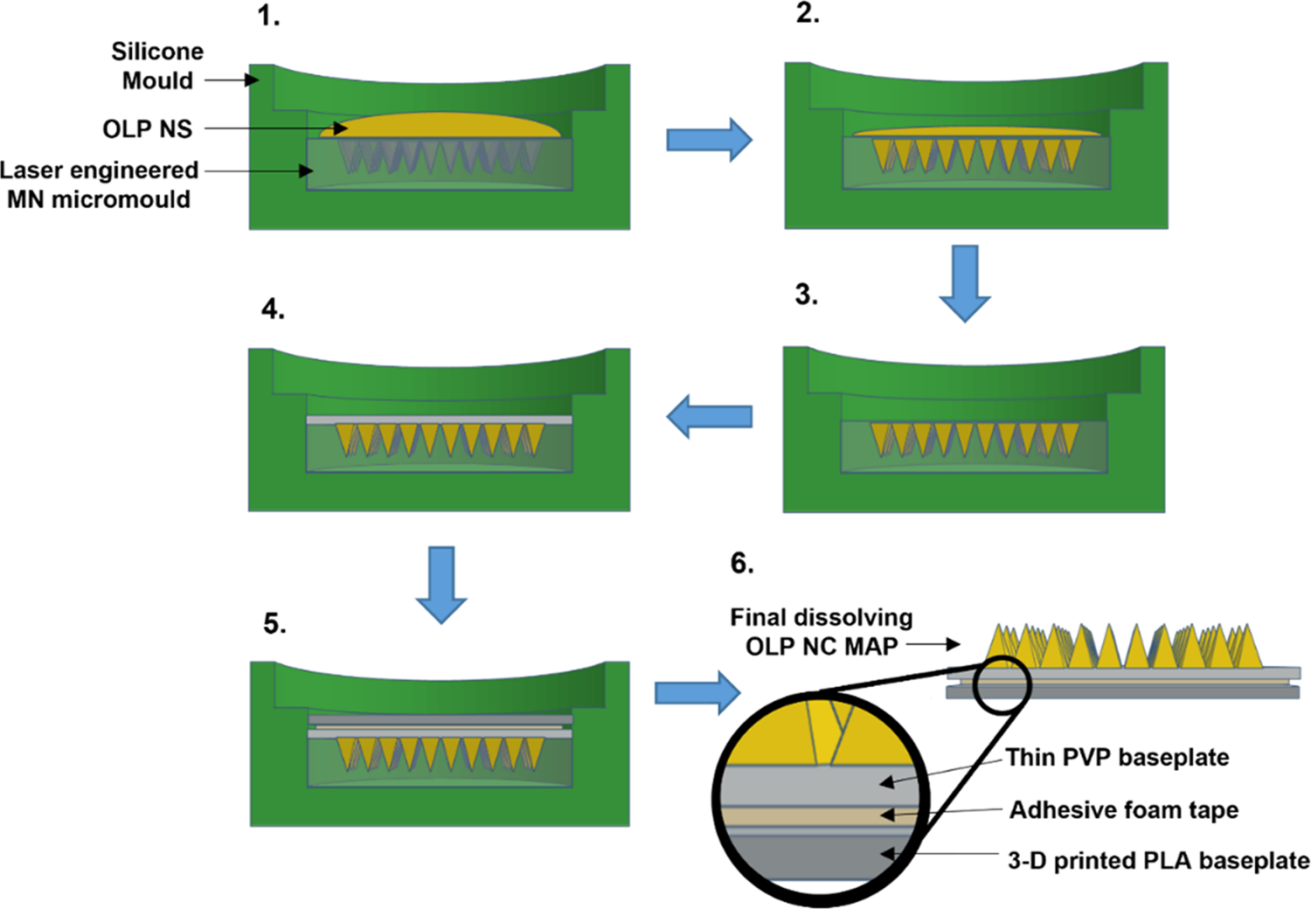
Dissolving olanzapine nanocrystal microarray patch preparation protocol—(1) olanzapine nanosuspension added to microarray patch mould, (2) Microneedle cavities filled using positive pressure, (3) excess olanzapine nanosuspension removed, and mould left to dry for 5 h, (4) poly(vinyl pyrrolidone) baseplate cast, centrifuged, and left to dry for 24 h, (5) three-dimensional (3D) printed poly(lactic acid) baseplate attached using adhesive foam tape, and (6) final microarray patch removed from mould.

Fabrication of dissolving MAPs containing OLP NCs located solely in the MNs, *i.e.*, not in the MAP baseplate, was achieved following reconstitution of OLP NC powder with deionized water in a ratio of 1:2 (100 mg of OLP NC powder reconstituted with 200 mg deionized water). The composition of OLP NC powder before and after reconstitution is presented in Table 4. To ensure the reconstituted powder was sufficiently mixed to produce a uniform OLP NS, it was placed inside a DAC 150 FVZ-K SpeedMixer (SpeedMixer, Lincoln Road, High Wycombe, U.K.), which was operated at 3500 RPM for 5 min. Immediately after this final mixing step, dissolving MAPs were prepared according to a protocol similar to that reported by Paredes et al. (depicted in Figure 2).^29^

**Table 4:** Composition of Olanzapine Nanocrystal Formulation before and after Reconstitution with Deionized Water

	concentration (% w/w)		
condition	OLP	PVA (MW = 9–10 kDa)	PVP (MW = 58 kDa)
before reconstitution	27.8	36.1	36.1
after reconstitution	9.3	12.0	12.0

Dissolving MAPs containing unprocessed OLP located solely in the MNs were also prepared following the same methodology; however, instead of using reconstituted OLP NS during MN casting, an aqueous blend of unprocessed OLP, PVA 9–10 kDa, and PVP 58 kDa with the same composition as stated in detail in Table 4 was used. A thin polymer baseplate was then cast by adding 200 μL of a 30% w/w aqueous solution of PVP 90 kDa to each MAP mould and centrifuging at 3000 RPM for 10 min. Moulds were left to dry at ambient laboratory conditions for 24 h. Finally, 3D printed PLA baseplates, prepared using the Ultimaker 3 3D printer (Ultimaker, Geldermalsen, Netherlands), were attached to the now solidified PVP baseplate using adhesive foam tape, and fully formed MAPs were then removed from their moulds by hand. The final MAPs produced were different from the hydrogel-forming arrays fabricated in Section 2.2.1 as they possessed 600 MNs, perpendicular to the base and of pyramidal shape (∼750 μm in height, with a base width of 300 μm, and 50 μm interspacing on a 0.7 cm^2^ array).

#### 2.3.5 Characterization of Dissolving MAPs

Characterization of the visual appearance, insertion profile, and mechanical strength of the formulated dissolving MAPs was carried out following the same procedures utilized for hydrogel-forming MN arrays as detailed in Sections 2.2.2.1 and 2.2.2.2. Additionally, the dissolution time and drug content of dissolving MAPs were calculated according to the same protocol used for OLP-containing DCTs in Section 2.2.4.1. Finally, to determine if formulation into MAPs adversely affected the particle size and PDI of OLP NCs, samples of dissolved OLP NC-containing MAPs were analyzed by DLS, as described previously.

#### 2.3.6 *In Vitro* Delivery of OLP from Dissolving MAPs

*In vitro* delivery of OLP from the formulated dissolving MAPs was investigated using the Franz cell apparatus according to the same protocol as outlined in Section 2.2.5. With dissolving MAPs, full-thickness neonatal porcine skin was used rather than dermatomed neonatal porcine skin, as it was deemed more representative of dissolving MAP-mediated delivery in an *in vivo* setting. As it was likely that OLP would be deposited within layers of the skin (epidermis and dermis) during *in vitro* delivery studies, it was imperative to ensure that OLP could be extracted from the skin in an effective manner. To do this, skin samples of ∼1 cm^2^ were cut into small pieces using a scalpel and then placed into 2 mL Eppendorf tubes. Each sample was then spiked with a known volume of OLP NS; in this case, 20 μL was used. As a control, 20 μL NS was added to vials without skin. Samples were vortexed for 30 s to mix OLP into and through the skin, centrifuged at 14,800 rpm for 1 min, and incubated in an oven at 37 °C for 24 h. Following incubation, two metal beads and 500 μL of deionized water were added to each vial and samples were homogenized in a tissue lyser at 50 Hz for 20 min. Once again, the samples were centrifuged at 14,800 rpm for 1 min, before 1 mL of ACN was added to each sample. Further homogenization at 50 Hz was carried out for 10 min, followed by sonication for 30 min. Finally, samples were centrifuged at 14,800 rpm for 10 min, and 50 μL of the supernatant was removed and diluted appropriately prior to analysis. Percentage recovery of OLP from neonatal porcine skin was calculated using eq 6
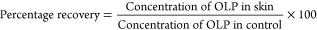
6

### 2.4 *In Vivo* Delivery of OLP from Hydrogel-Forming and Dissolving MAPs

**Figure 3:**
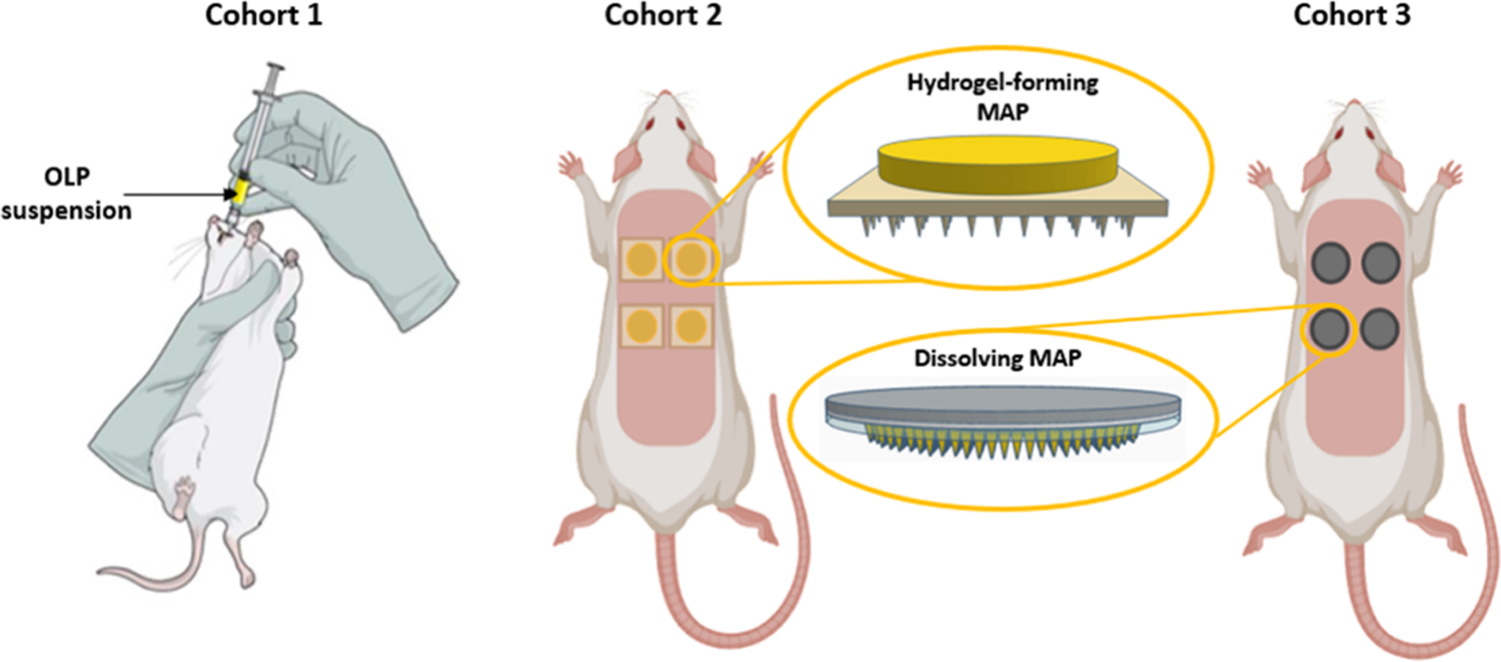
Schematic representation of doses administered to animals in each cohort.

Delivery of OLP from the previously formulated OLP MAPs, namely, CD inclusion complex-containing hydrogel-forming MAPs (PVA/PVP hydrogel-forming MN arrays with PEG.SD DCTs) and NC-containing dissolving MAPs, in an *in vivo* setting was investigated using healthy female Sprague-Dawley rats (*n* = 18) aged 10–12 weeks and weighing 259 ± 19 g. Animals were acclimatized to laboratory conditions for a minimum of 7 days prior to the experiment. Rats were divided into three cohorts (*n* = 6 per cohort), the first of which was the control cohort that received OLP orally in the form of an oral suspension, as the most commonly prescribed form of OLP is standard, immediate release tablets.^30^ The remaining cohorts each received OLP *via* the previously formulated MAPs, with CD inclusion complex-containing hydrogel-forming MAPs applied to animals in cohort two and dissolving NC-containing MAPs applied to those in cohort three. After 24 h, MAPs were carefully removed from rats in these cohorts. Precise details of the doses received by each cohort are presented in Table 5 and Figure 3. Blood samples were taken *via* tail vein bleed at predefined time intervals of 1, 2, 4, 6, 24, 48, and 72 h, with ∼200 μL collected at each sampling point. At the end of the experiment, animals were humanely culled using a CO_2_ chamber followed by cervical dislocation. This study was approved by the Committee of the Biological Services Unit of Queen’s University Belfast. Experimentation was conducted under Procedure Project Licence number 2903 and Procedure Individual Licence numbers 1892, 2058, and 2059 according to the policy of the Federation of European Laboratory Animal Science Associations and the European Convention for the protection of vertebrate animals used for experimental and other scientific purposes with implementation of the principles of 3Rs (replacement, reduction, and refinement).

**Table 5:** Details of Doses Administered to Animals in Each Cohort

cohort	dosing method	total dose administered (mg/rat)	total dose administered (mg/kg)
1	1 mL of OLP suspension (2.5% w/w) *via* oral gavage	2.5	10
2	4× hydrogel-forming MAPs	5.0	20
3	4× dissolving MAPs	5.0	20

Following collection *via* tail vein bleed, blood samples were centrifuged at 2000*g* at 4 °C for 10 min to separate plasma from blood. Plasma was then collected and stored at −20 °C until analyte extraction. To extract OLP from plasma, 900 μL of ACN was added to 100 μL of OLP-containing plasma in a 1.5 mL Eppendorf tube and then vortexed at high speed for 10 s to ensure adequate mixing of the two phases. Samples were then vortexed further at a high speed for 10 min before centrifugation at 14,000 RPM at 4 °C for 10 min. Sample supernatant was collected and filtered through a 0.2 μm PTFE syringe filter into a disposable glass culture tube and dried under a stream of nitrogen at 35 °C for 40 min using a Zymark TurboVap LV Evaporator Workstation (McKinley Scientific, Sparta, NJ). The dried residue was then reconstituted in 100 μL of H_2_O containing 50% v/v ACN and vortexed at a low speed for 30 s to ensure complete sample solvation. Finally, the reconstituted sample was transferred to an Agilent HPLC vial (with 250 μL insert in place) in preparation for analysis using HPLC-UV.

#### 2.4.1 Calculation of pharmacokinetic parameters and relative bioavailability

Following pharmaceutical analysis, noncompartmental pharmacokinetic parameters of the delivery profiles obtained for each cohort, namely, the maximum observed plasma concentration (*C*_max_) of OLP and the time at which the *C*_max_ was observed (*T*_max_), were determined by inspection of the raw data. Additionally, total OLP exposure, which was represented by the area under the curve (AUC) of each delivery profile, was calculated using the linear-log trapezoidal method. Bioavailability of a compound is described as the fraction of the administered dose that reaches the systemic circulation.^31^ Bioavailability (*F*) of a therapeutic can be calculated using eq 7, where clearance (Cl), AUC, and dose are all important factors
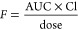
7Relative bioavailability is a pharmacokinetic parameter used to compare the bioavailability of a drug when administered as part of a given formulation or *via* a specific route, with the bioavailability of the same drug when administered as part of a different formulation or *via* an alternative route.^31^ In this work, the relative bioavailability of OLP when administered by each type of MAP was calculated in relation to the bioavailability of OLP when administered *via* the oral route, which is commonly reported in the literature as 57%.^32−34^ Regardless of the route of administration, the clearance of OLP in all cohorts was considered to be the same. With this established, when eq 7 is rearranged to find clearance, eq 8 is obtained
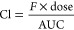
8To calculate an estimate of the relative bioavailability of OLP administered *via* either MAP type (*F*^MAP^), the clearance equation of the MAP-treated group was set equal to the clearance equation of the orally treated group to get eq 9
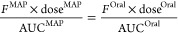
9Finally, eq 9 was rearranged to solve for *F*^MAP^ to obtain eq 10, where the oral bioavailability of OLP (*F*^Oral^) was considered to be 57%^32−34^
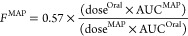
10

### 2.5 Pharmaceutical Analysis

Analysis of samples from *in vitro* experimentation was realized on an Agilent 1200 system (Agilent Technologies U.K. Ltd., Stockport, Greater Manchester, U.K.) using a Waters XSelect CSH C18 column (150 mm × 3 mm internal diameter, 3.5 μm packing) (Waters Corporation, Dublin, Ireland) with UV detection at 225 nm (run time = 7 min). Mobile phase composition for this isocratic separation method was 40:60 0.01 M ammonium acetate buffer (pH 8.6, adjusted with ammonium hydroxide 1 M: ACN at a flow rate of 0.3 mL/min). The sample solvent was PBS containing 50% v/v ACN, the column temperature was maintained at 20 °C, and each injection for analysis had a volume of 20 μL. To successfully analyze samples from *in vivo* experimentation, this method was altered to obtain a gradient separation method that provided adequate resolution of OLP from components of rat plasma. The column type, UV detector wavelength, and mobile phase composition were kept the same. The mobile phase composition was adjusted according to the ratios in Table 6. Flow rate and column temperature were maintained at 0.5 mL/min and 40 °C, respectively, and each injection for analysis had a volume of 60 μL. To ensure a high level of accuracy throughout the analysis, plasma standards, which contained a known concentration of OLP, were analyzed before, during, and after analysis of study samples. Agilent ChemStation Software B.02.01 was used for chromatogram analysis for all samples tested.

**Table 6:** Mobile Phase Composition during Gradient Elution in the UV-HPLC Method for *In Vivo* Sample Analysis

time (min)	*A*%	*B*%
0.0	90.0	10.0
2.0	90.0	10.0
2.3	29.0	71.0
8.0	29.0	71.0
10.0	10.0	90.0
11.0	90.0	10.0
16.0	90.0	10.0

### 2.6 Statistical Analysis

Statistical analysis was performed using GraphPad Prism 7 (GraphPad Software, San Diego, CA). Where appropriate, an unpaired *t*-test was used for comparison of two groups. A one-way analysis of variance (ANOVA) was used for the comparison of multiple groups. In all cases, data was presented as either the mean ± standard deviation (S.D.) or the mean + S.D., and *p* < 0.05 denotes statistical significance.

## 3 Results and Discussion

### 3.1 Hydrogel-Forming MAPs

#### 3.1.1 Characterization of Hydrogel-Forming MN Arrays

**Figure 4:**
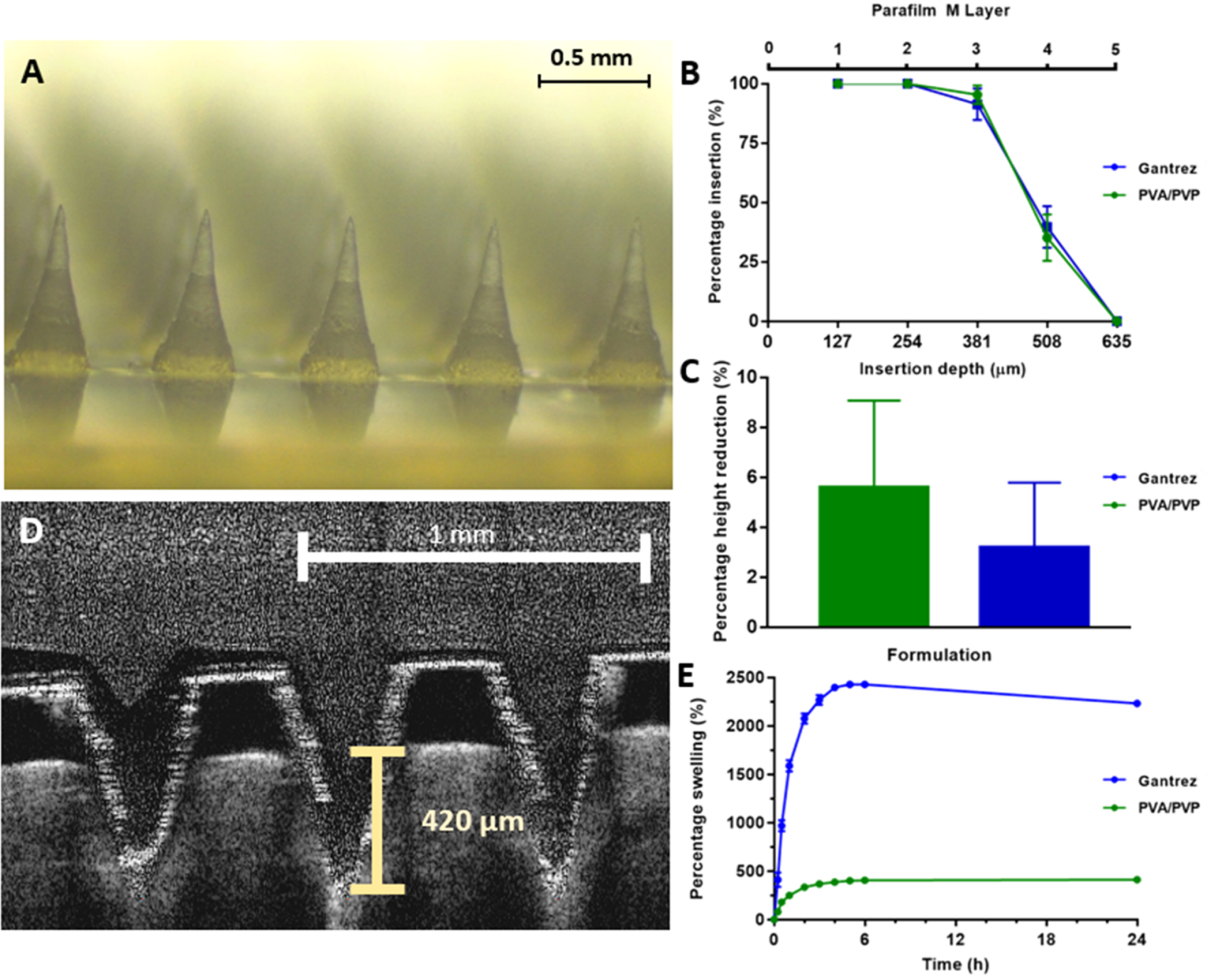
(A) Representative image of formulated hydrogel-forming microneedle arrays obtained using a digital light microscope, (B) percentage insertion and estimated insertion depth and (C) percentage microneedle height reduction of both hydrogel-forming microneedle types following insertion into an artificial skin model at a force of 32 N. (D) Exemplar optical coherence tomography image of a hydrogel-forming microneedle array inserted into neonatal porcine skin. (E) Swelling profiles of Gantrez and PVA/PVP hydrogels submerged in phosphate-buffered saline (pH 7.4 ± 0.1) over 24 h. Where appropriate, data is presented as the mean + or ± S.D., *n* = 5.

A representative digital light microscope image of the formulated hydrogel-forming MN arrays is presented in Figure 4A, with the percentage insertion and estimated insertion depth of MNs into an artificial skin model following an application force of 32 N presented in Figure 4B. Both MN types achieved 100% insertion into the first two layers of Parafilm M, which equates to an insertion depth of ∼254 μm. High levels of insertion were also observed in layer three where 95.3 ± 3.9 and 91.4 ± 6.6% of MNs had inserted for Gantrez and PVA/PVP formulations, respectively. A reduced insertion efficiency was recorded in layer four (insertion depth ∼ 508 μm) where the percentage MN insertion was 35.2 ± 9.8 and 39.7 ± 8.8%, respectively. Neither formulation penetrated the fifth layer of Parafilm M, as the total membrane thickness at this depth exceeded the height of the formulated MNs. In their dry (xerogel) state, the formulated MNs demonstrated their ability to successfully penetrate the outermost layers of the skin upon application of a force similar to that applied by the hand of a patient.

Following insertion, the percentage MN height reductions observed for Gantrez and PVA/PVP formulations were 5.6 ± 3.5 and 3.2 ± 2.6%, respectively (Figure 4C). These results highlight the acceptability in terms of the mechanical strength of hydrogel-forming MNs prepared from each of the formulations tested. The insertion depths of MNs into full-thickness neonatal porcine skin were 438 ± 15 and 427 ± 11 μm for Gantrez and PVA/PVP hydrogel-forming MNs, respectively (Figure 4D). These values were consistent with the insertion depth values reported previously^20^ and highlight the suitability of the artificial skin membrane protocol to accurately assess the insertion depth into neonatal porcine skin.^18^ The Gantrez formulation had a significantly greater swelling capacity than the PVA/PVP formulation (*p* < 0.0001). After 24 h, the percentage swelling of the Gantrez hydrogel was 2231.9 ± 27.3%, whereas the PVA/PVP hydrogel had swollen by 410.9 ± 17.2% (Figure 4E). Furthermore, the Gantrez formulation demonstrated a more rapid swelling profile than the PVA/PVP formulation. This is well illustrated by the results obtained 15 min after submersion in PBS where the observed percentage swellings of each formulation were 411.4 ± 73.8 and 79.4 ± 17.6%, respectively. During manufacture, the polymers in each of the hydrogel-forming array formulations tested here are thermally cross-linked, through ester bond formation, to produce insoluble, yet swellable polymer matrices. The occurrence of these ester bonds, also referred to as the degree of cross-linking or cross-link density, within a hydrogel network directly influences its swelling ability, *i.e.*, increased cross-link density results in reduced swelling and *vice versa*. As such, the functionality and molecular weight of both the polymer and cross-linker used in a hydrogel formulation play an important role in cross-linking and, therefore, swelling that, ultimately, influences drug delivery *via* hydrogel-forming MAPs.^35,36^ Considering the hydrogels tested here, the PVA/PVP formulation demonstrated reduced swelling, compared to the Gantrez formulation, due to the higher cross-link density that exists within its polymer network.^20^ In the PVA/PVP formulation, cross-linking occurs in the form of esterification between the hydroxy group present on the PVA and carboxylic acid groups present on citric acid.^17^ Citric acid is an effective cross-linker as it has a high number of reactive carboxylic groups relative to its molecular weight, or unit mass, which increases the frequency at which ester bonds are formed with PVA. Furthermore, citric acid has a small molecular weight (192 Da) that reduces the distance between cross-linked PVA molecules, thus reducing the size of pores present in the hydrogel matrix. These factors serve to increase cross-link density within a hydrogel and result in a rigid polymer network with reduced overall swelling capacity.^17,20^ In the Gantrez formulation, cross-linking is also achieved through an esterification reaction between the polymer and the cross-linker; however, in this case, ester bonds are formed between carboxylic groups on Gantrez S-97 (polymer) and terminal hydroxy groups on PEG 10,000 (cross-linker).^37^ This hydrogel demonstrated increased swelling due to a reduced cross-link density within its polymer network. This reduction in cross-link density can be attributed to the inclusion of sodium carbonate in this formulation that reduces ester bond formation by interacting with free carboxylic acid groups on Gantrez S-97 to produce sodium salts, thus reducing the frequency at which cross-linking occurs.^16^ Additionally, PEG 10,000 has a low number of reactive hydroxy groups relative to its unit mass, which further limits ester bond formation with the polymer.^35^ Finally, the higher molecular weight of PEG 10,000, compared to citric acid, increases the distance between cross-linked Gantrez S-97 molecules, increasing the pore size within the polymer network and enhancing the extent to which this hydrogel can absorb and retain water.^38^

#### 3.1.2 OLP/CD Phase-Solubility Analysis

**Figure 5:**
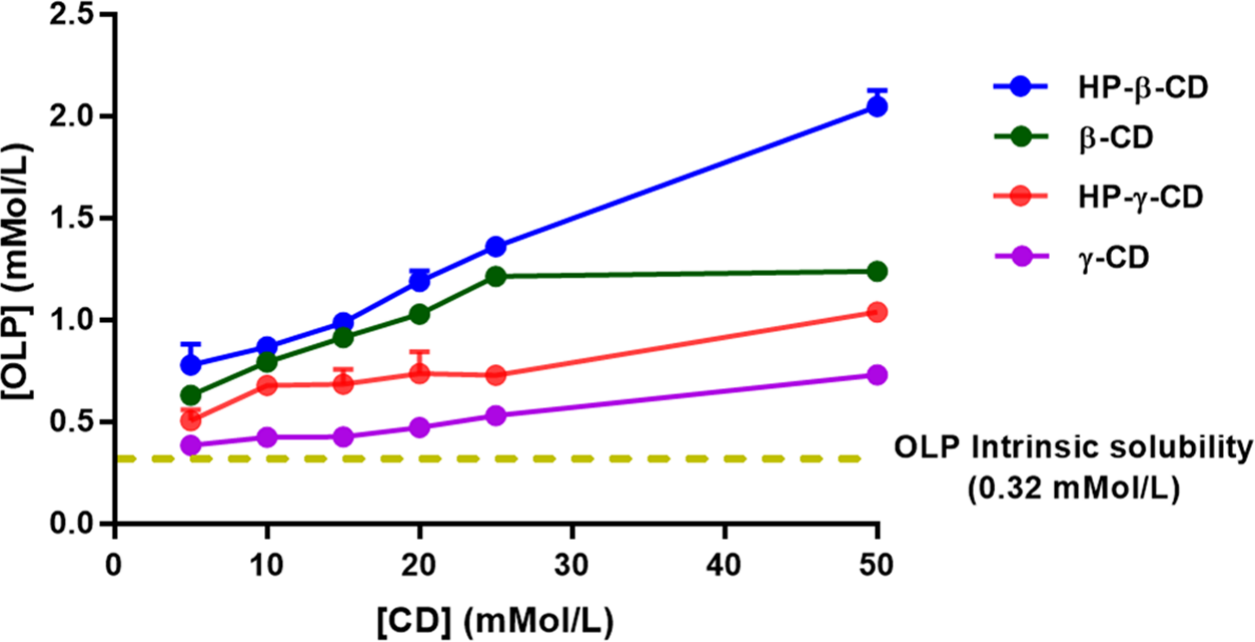
Phase-solubility diagram of olanzapine in PBS (pH 7.4) containing increasing concentrations of β-CD, HP-β-CD, γ-CD, or HP-γ-CD over 24 h (37 ± 1 °C and 40 RPM) (mean + S.D., *n* = 3).

Phase-solubility studies were carried out to investigate the ability of β-CD, HP-β-CD, γ-CD, or HP-γ-CD to complex with and improve the aqueous solubility of OLP (Figure 5). In all cases, the observed aqueous solubility of OLP was enhanced by the addition of CD. For all CDs tested, maximum OLP solubility enhancement was observed at the highest concentration of CD, *i.e.*, 50 mMol/L (Table 7).

**Table 7:** Observed Concentration of Olanzapine in PBS Containing Different Cyclodextrins at 50 mMol/L (*t* = 24 h, 37 ± 1 °C, and 40 RPM) (mean ± S.D., *n* = 3)

sample composition	OLP concentration (mMol/L)	OLP concentration (μg/mL)
OLP	0.32 ± 0.01	100.05 ± 2.63
OLP + γ-CD	0.73 ± 0.02	228.15 ± 6.06
OLP + HP-γ-CD	1.04 ± 0.03	325.40 ± 10.07
OLP + β-CD	1.24 ± 0.02	387.13 ± 7.47
OLP + HP-β-CD	2.05 ± 0.08	639.63 ± 24.92

Complexes formed between OLP and HP-β-CD displayed the greatest enhancement of OLP solubility, a 6.39-fold increase. This was followed by native β-CD that attributed a 3.83-fold increase in the aqueous solubility of OLP. HP-γ-CD and its native counterpart γ-CD provided reduced levels of solubility enhancement, with 3.20- and 2.24-fold improvements, respectively. This may be attributed to their large cavity diameter (∼7.5 Å) in comparison to the low MW of OLP (312.44 g/mol).^39^ In such instances, both the frequency and extent of interactions between the hydrophobic cavity of a CD and the hydrophobic drug molecule are low, and reduced solubility enhancement is observed. β-CD possesses a smaller cavity diameter compared to that of γ-CD (∼6.0 Å).^39^ Its performance was statistically superior to that of γ-CD (*p* = 0.0043), which indicated an increased compatibility with OLP. A CD must be in solution in order to interact with and house a drug molecule within the internal cavity of its cyclic structure.^40^ The aqueous solubilities of HP-β-CD and β-CD are reported to be >500 and 18.5 mg/mL, respectively.^40^ The improved solubility of HP-β-CD is attributed to derivatization of native β-CD molecules with hydroxypropyl groups that interact with water molecules in an extensive manner.^26,40,41^ The concentration of OLP observed when in the presence of HP-β-CD (50 mMol/L) was 639.63 ± 24.92 μg/mL. This was significantly larger than the concentration of OLP observed in the presence of native β-CD at the same concentration (*p* < 0.0001) and can be explained by the increased solubilization capacity of HP-β-CD, due to its greater aqueous solubility. OLP concentration increased linearly as a function of CD concentration in the presence of HP-β-CD, which is characteristic of an A_L_-type phase-solubility profile and, when combined with a slope value below unity (<1), indicates first-order inclusion complex formation, *i.e.*, 1:1 molar OLP-CD stoichiometry.^22,42^

#### 3.1.3 Characterization of OLP/CD Inclusion Complexes

##### 3.1.3.1 DSC, PXRD, FTIR, SEM, and Percentage Yield

**Figure 6:**
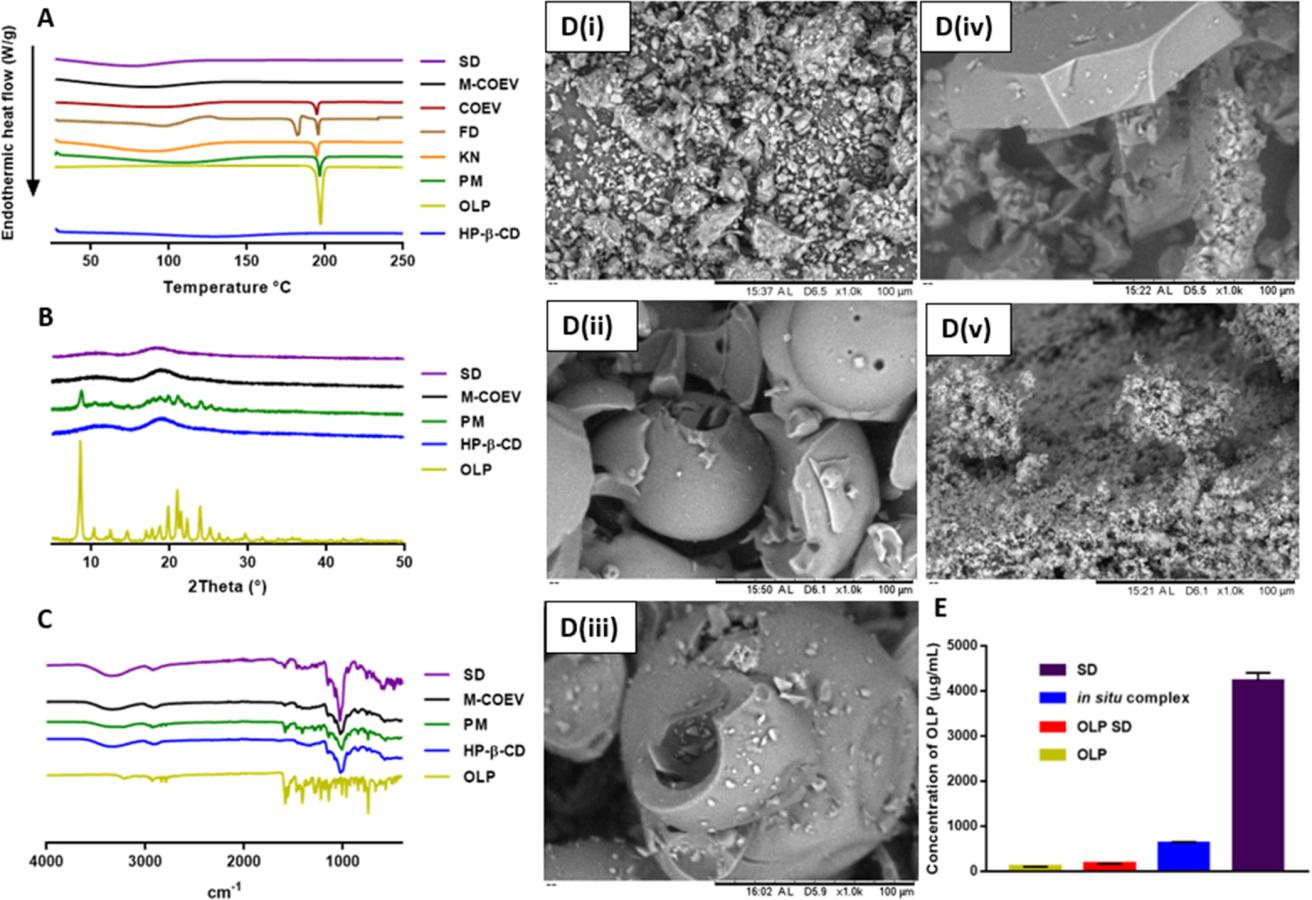
Traces obtained from the analysis of HP-β-CD (blue line), OLP (light green line), a PM of OLP, and HP-β-CD (blue line), inclusion complexes formed by the processes of KN (orange line), FD (tan line), COEV (red line), M**-**COEV (black line), and SD (purple line) using (A) DSC, (B) PXRD, and (C) FTIR. Additionally, (D) SEM photomicrographs of (i) OLP, (ii) HP-β-CD, and (iii) PM of OLP and HP-β-CD and OLP/HP-β-CD inclusion complexes formed by (iv) M-COEV and (v) SD. Finally, (E) maximum observed concentration of OLP (μg/mL) in PBS for samples containing OLP, OLP processed by SD, OLP/HP-β-CD inclusion complexes formed *in situ* and OLP/HP-β-CD complexes formed in the solid state by SD (mean + S.D., *n* = 3).

A broad endothermic peak at 80–120 °C attributed to the dehydration of CD molecules was observed on the DSC trace for HP-β-CD^43^ (Figure 6A). Additionally, the absence of a sharp endothermic peak on the trace for HP-β-CD indicated that a crystalline structure was not present within the sample tested.^44^ Analysis of pure OLP presented a sharp peak at ∼198 °C. This peak corresponds to the breakdown of OLP’s crystalline structure, *i.e.*, the melting point of OLP.^44^ Thermal analysis of PM provided a trace similar to that of a combination of HP-β-CD and OLP. A minimal shift of the peak associated with the melting point of OLP to a lower temperature illustrated the slight interaction between HP-β-CD and OLP that most likely occurred during either PM preparation or the thermal analysis process.^43,44^ A reduction in the intensity of the OLP melting peak was caused by the reduced concentration of OLP in these samples compared to pure drug samples.^43,44^ Analysis of solid-state inclusion complexes formed by KN, FD, and COEV displayed OLP melting peaks that were broader and that had shifted further toward a lower temperature. This finding is typical of the increased amorphous content and is attributed to partial inclusion of OLP inside HP-β-CD molecules.^43,44^ Interestingly, the thermogram obtained for inclusion complexes formed by FD had an additional endothermic peak at ∼185 °C. Possible causes of this new peak appearance include formation of OLP with an alternative crystalline structure, or CDs aggregating to form crystalline lattice-type structures.^45^ Further work will investigate the true origin of this new peak. The DSC trace of OLP/HP-β-CD inclusion complexes formed by M-COEV and SD revealed complete disappearance of the OLP melting peak. This peak disappearance was attributed to the transition of OLP from a crystalline to an amorphous state by complete inclusion inside HP-β-CD molecules.^43,44,46^

PXRD analysis of OLP (Figure 6B) revealed an intense peak at a diffraction angle of 2θ equal to 8.62°. Moreover, a series of similar, yet less intense, peaks between 14.58 and 27.42° were observed. The presence of these peaks confirmed the crystalline structure of OLP.^43,44,46^ Analysis of HP-β-CD using PXRD gave a diffractogram with no visible crystalline peaks and two broad peaks of low intensity. These observations are typical of an amorphous substance.^24,44^ As with the findings from DSC analysis, the trace associated with PM was comparable to an overlapping of drug and CD traces. No crystalline peaks were observed in the diffractograms of M-COEV and SD, with the amorphous nature comparable to pure HP-β-CD displayed.

FTIR analysis of pure OLP showed a band at approximately 3400–3100 cm^–1^ corresponding to the amino group of the ring structure containing two nitrogen atoms (Figure 6C).^44^ The spectrum obtained for HP-β-CD contains a band at approximately 3600–2950 cm^–1^, which was consistent with the considerable amount of free OH groups within the structure of the molecule.^44,47^ An overlap of the two bands mentioned previously can be seen in the spectrum of PM. The absence of a band shift in this case indicated the lack of chemical interaction between the two substances when they coexist in a physical mixture.^44^ When the spectra of M-COEV and SD were analyzed, band shifts from 3339.9 to 3337.1 cm^–1^ and 3337.6 cm^–1^, respectively, and from 2967.7 to 2969.3 cm^–1^ and 2971.1 cm^–1^, respectively, are observed. These displacements suggest hydrogen bonding between hydroxyl groups on HP-β-CD and the previously described amino group on OLP.^44,47^ Similarly, peak shifting in the form of N–H bending was identified by peak shifts from 1583.3 to 1588.3 cm^–1^ and 1589.4 cm^–1^, which further reinforces the importance of hydrogen bonding in inclusion complex formation.^47^ Finally, the spectra for M-COEV and SD displayed peak shifts in the region of 725–760 cm^–1^, which is consistent with C–H bending of 1,2-substituted arene compounds.^48^ This finding implied complexation of the aromatic ring on OLP inside the hydrophobic cavity of HP-β-CD.

Following SEM analysis, pure OLP (Figure 6D(i)) appeared as relatively small, irregularly shaped crystals that displayed instances of self-agglomeration. Alternatively, HP-β-CD (Figure 6D(ii)) existed as larger, hollow, spherical structures typical of amorphous substances.^44^ In agreement with DSC, PXRD, and FTIR analyses, SEM images of PM (Figure 6D(iii)) displayed small OLP crystals in the presence of amorphous HP-β-CD with minimal interaction between the two materials. Analysis of binary mixtures of OLP and HP-β-CD processed by M-COEV and SD (Figure 6D(iv and v)) exhibited a conversion of OLP’s original crystalline morphology into one that was amorphous in nature. Samples obtained by M-COEV existed as small, irregularly shaped particles that had aggregated extensively to form larger and increasingly nonuniform structures. This observation may be attributed to the nature of complex collection, *i.e.*, the action of scraping the evaporated complex from the inside of the round-bottom flask. SEM photomicrographs of SD samples illustrated small, amorphous particles that displayed increased uniformity in both size and morphology when compared to M-COEV samples. Accordingly, this finding also may be attributed to the use of a nebulizer with defined pore size and the method of complex collection following the spray-drying process, wherein the product emerges from the spray-drier head as a dry powder and is easily collected from the inner surface of the spray-drier drum. The percentage yield of solid-state OLP/HP-β-CD inclusion complexes obtained from M-COEV was 54.1 ± 4.7%, which was statistically less than that of the SD method (73.6 ± 2.7%, *p* < 0.0001). This finding confirmed that CD complexation *via* organic solvent spray-drying was more efficient than organic solvent coevaporation (M-COEV) in terms of the yield of the powdered product obtained. If upscaling of the production of OLP/HP-β-CD inclusion complexes were to be required, it is likely that this would be the chosen complexation method. Drug content of this powdered product was indirectly confirmed during DCT characterization as detailed in Section 3.1.4. Future work should include determination of residual solvent content of the powdered product obtained from this process, with particular reference to acceptable levels for transdermal patch-type products.

##### 3.1.3.2 Solubility Enhancement Inferred by CD Complexation

The intrinsic solubility of OLP in PBS was calculated to be OLP 100.05 ± 2.63 μg/mL (Figure 6E). To deduce the extent to which the process of spray-drying alters the solubility of OLP, the drug was spray-dried in the absence of HP-β-CD. After 24 h (37 ± 1 °C and 40 RPM), the observed solubility of OLP SD in PBS was 163.11 ± 4.40 μg/mL. This relatively minimal increase in OLP solubility can be attributed to the breakdown and subsequent reformation of OLP’s crystalline structure during spray-drying. However, the extent of solubility enhancement contributed by the SD process alone was negligible in comparison to the effect of CD complexation *in situ* (*p* < 0.0001). Considering OLP/HP-β-CD complexes formed by the novel process of spray-drying in methanol, upon dissolution in PBS (*t* = 1 min), a greatly increased OLP concentration of 4217.85 ± 183.96 μg/mL was observed. This large increase in OLP solubility was attributed to an increased level of complexation being achieved by the dissolution of OLP and HP-β-CD in methanol followed by rapid solvent removal *via* spray-drying. At *t* = 15 min, the concentration of OLP observed in the same samples had fallen to 548.79 ± 61.86 μg/mL, where they remained for the duration of the study (concentration of OLP at *t* = 24 *h* = 556.26 ± 47.06 μg/mL). This was statistically similar to the concentration of OLP observed at the same timepoint with complexes formed *in situ* (*p* = 0.0768). The observed spring in OLP concentration followed by a substantial reduction at 15 min can be explained by the high-energy state in which these amorphous, solid-state complexes exist. Upon dissolution, these complexes exhibit very high aqueous solubility. However, this data suggests that the same complexes are unable to maintain the high-energy form obtained by spray-drying in methanol and rapidly dissociate in solution.

#### 3.1.4 Characterization of Formulated DCTs

All formulations produced DCTs that had consistent average masses and, therefore, passed the BP uniformity of mass test (Table 8). In a similar manner, drug recovery from all DCTs tested was deemed acceptable with average values ranging between 98.34 and 99.12%. There was little variation between the diameters of DCTs produced from all formulations. However, FSD and FPEG.SD DCTs had significantly reduced thicknesses when compared to FS, FCD, and FX DCTs (*p* < 0.05 in all cases). This can be attributed to the fact that the product of the spray-drying process is a feathery and easily compactable powder composed of uniformly small particles, whereas the components that make up FS, FCD, and FX DCTs are coarse and nonuniform in size and shape. This increased level of compaction resulted in less penetration of water into the structure of the tablet and, therefore, more time being required for these DCTs to dissolve. Considering tablet hardness, a direct comparison can be made between FX and SD DCTs as the only difference between the two formulations is that in SD, OLP and HP-β-CD have been processed *via* spray-drying. The hardness of SD DCTs was 32.20 ± 4.15 N, and this was significantly harder than FX DCTs, which had a hardness of 23.25 ± 4.86 N (*p* = 0.0264). This further supports the hypothesis that a greater level of compaction can be achieved with a spray-dried powder compared to a powder that is unprocessed. Evidently, the same cannot be said for SD.PEG DCTs; however, the reduced hardness of these tablets can be explained by the inclusion of PEG 3400 in this formulation, which exists as a waxy solid that is readily friable at ambient laboratory conditions. From a translational point of view, this may be an aspect of this formulation that requires further optimization; however, it could be argued that adequate packaging may avoid and downstream user issues. All DCT formulations demonstrated adequate dissolution times when submerged in aqueous media. FX DCTs contained the insoluble super disintegrant CPV that, of course, did not dissolve but aided the disintegration of the soluble components of these DCTs and, therefore, sped up their dissolution time.

**Table 8:** Physical Characteristics of Directly Compressed Tablets Composed of FS, FCD, FX, SD, and PEG.SD Formulations (Mean ± S.D., *n* = 5)

parameter	FS	FCD	FX	SD	PEG.SD
diameter (mm)	10.02 ± 0.02	10.01 ± 0.02	10.04 ± 0.05	9.97 ± 0.01	9.98 ± 0.01
thickness (mm)	1.13 ± 0.01	1.11 ± 0.02	1.10 ± 0.02	0.97 ± 0.02	0.93 ± 0.02
hardness (N)	21.56 ± 5.31	27.69 ± 4.12	23.25 ± 4.86	32.20 ± 4.15	18.60 ± 2.61
average mass (mg)	99.12 ± 1.08	98.89 ± 0.77	98.34 ± 0.94	98.58 ± 0.96	98.22 ± 1.02
uniformity of mass test result	PASS	PASS	PASS	PASS	PASS
dissolution time (s)	42.05 ± 1.76	68.11 ± 2.73	40.71 ± 0.57	70.00 ± 6.12	95.20 ± 8.81
drug recovery (%)	98.95 ± 0.97	99.12 ± 1.26	99.21 ± 1.48	98.64 ± 1.32	98.52 ± 1.25

#### 3.1.5 *In Vitro* Delivery of OLP from Hydrogel-Forming MAPs

**Figure 7:**
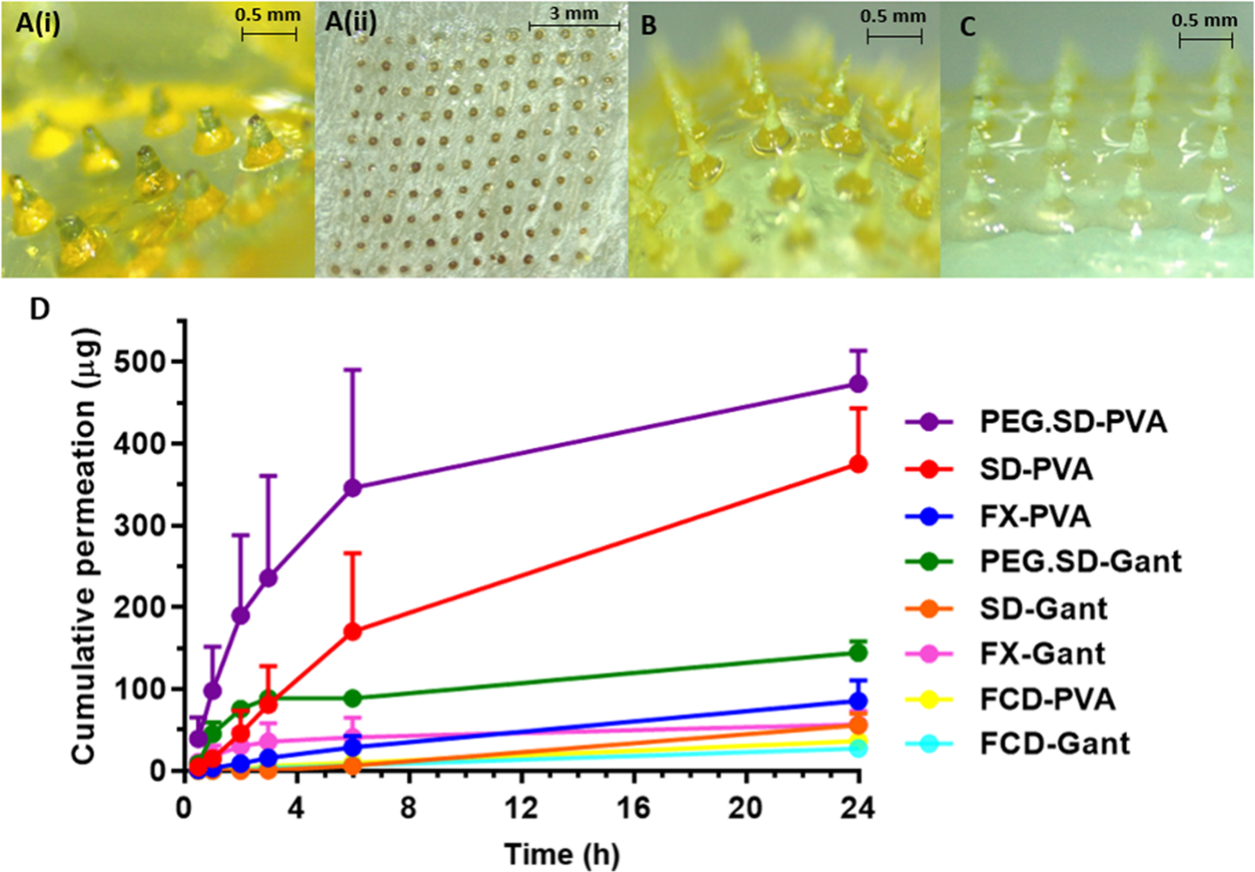
Representative images following *in vitro* permeation testing of (A(i)) FCD directly compressed tablet with a Gantrez hydrogel-forming MN array with tips detached and (A(ii)) corresponding dermatomed neonatal porcine skin with implanted microneedle tips (*t* = 24 h). (B) FCD and (C) PEG.SD directly compressed tablet with a PVA/PVP hydrogel-forming MN array with tips intact (*t* = 24 h). (D) Cumulative permeation of olanzapine from all directly compressed tablet formulations across dermatomed neonatal porcine skin *via* hydrogel-forming microneedle arrays using the Franz cell apparatus (mean + S.D., *n* = 5).

FS DCTs were tested in combination with hydrogel-forming MN arrays and without (*i.e.*, DCTs applied directly to skin). In replicates where hydrogel-forming MN arrays were absent, DCTs did not dissolve and OLP was not detected in the receiver compartment of the Franz cell apparatus over the course of 24 h. In replicates where hydrogel-forming MN arrays were inserted into the skin prior to DCT application, FS DCTs had fully dissolved after 24 h. However, once again, OLP was not detected in the receiver compartment at any point during the 24 h study for either array type. This was attributed to the inability of the hydrophobic drug OLP to permeate through the aqueous environment of a swollen hydrogel MN array. In a similar fashion, FCD DCTs were tested in the absence of MN arrays, and in the presence of both types of hydrogel-forming MN array. In replicates where MN arrays were not present, FCD DCTs displayed only slight dissolution and, consequently, no permeation of OLP across dermatomed neonatal porcine skin. These results were similar to those observed with FS DCTs and indicated that the addition of CDs alone, *i.e.*, without the use of MN arrays, was insufficient to permit transdermal permeation of the poorly soluble drug OLP. When hydrogel-forming MN arrays were inserted into the skin before FCD DCT application, FCD DCTs demonstrated complete dissolution, and transdermal permeation of OLP into the receiver compartment was achieved. After 24 h, the cumulative permeation values of OLP from FCD DCTs were 27.32 ± 8.59 μg (3.94 ± 1.24%) and 36.32 ± 9.76 μg (5.24 ± 1.41%) for Gantrez and PVA/PVP MN arrays, respectively. An interesting observation was that the tips of Gantrez MNs had detached and were implanted in the skin following MN array removal (Figure 7A(i and ii)), whereas those of PVA/PVP MNs remained fully intact (Figure 7B). This was not observed with FS DCTs, and it was postulated that the external hydroxy groups on the structure of HP-β-CD may have interacted with hydrogel components, *i.e.*, Gantrez S-97 or PEG 10,000, causing the hydrogel to become structurally weakened, leading to detachment of MN tips in the skin. Further work is required to identify the true nature of this occurrence.

To study the effect that excipient use had on OLP permeation, FX DCTs were tested using both types of hydrogel-forming MN arrays. In both cases, after 24 h, FX DCTs had completely dissolved and the cumulative permeation values of OLP observed were 56.46 ± 16.84 μg (8.15 ± 2.43%) and 85.66 ± 25.36 μg (12.36 ± 3.66%) for Gantrez and PVA/PVP MN arrays, respectively. The transdermal drug permeation observed here was statistically greater than that in FCD DCTs in all cases (*p* < 0.05) except for the comparison between FCD-PVA and FX-Gant (*p* = 0.1123). In FCD replicates, quantifiable levels of OLP were not detected in the receiver compartments of both hydrogel types until the 2 h timepoint, whereas in FX replicates, OLP was at a quantifiable level after 30 min. The reduced permeation time and increased overall permeation observed were attributed to the rapidly dissolving nature of FX DCTs, and, therefore, the ability of the excipients used to enhance the DCT dissolution rate. As before, swollen hydrogel arrays were yellow in color due to the presence of OLP in their matrix and the tips of Gantrez MNs had detached, whereas the tips of PVA/PVP MNs remained intact.

Solid-state OLP/HP-β-CD inclusion complexes formed by spray-drying were formulated into SD and PEG.SD DCTs and the *in vitro* permeation of OLP from these DCTs and was investigated. After 24 h, complete dissolution of all DCTs tested was confirmed upon apparatus disassembly. Considering SD DCT replicates, the cumulative permeation using Gantrez MN arrays was 55.53 ± 14.14 μg (8.01 ± 2.04%) after 24 h. This was not statistically greater than that observed with FX DCTs using the same hydrogel-forming MN array type, which revealed that spray-drying inclusion complexes provided no additional benefit in this instance. At the same timepoint, the cumulative permeation of OLP from PEG.SD DCTs using the Gantrez MN arrays was 144.51 ± 13.64 μg (20.85 ± 1.97% permeation), which was significantly greater than the permeation observed with FX DCTs (*p* = 0.0021). This finding indicated that, by forming inclusion complexes through spray-drying in the presence of PEG 3400, the transdermal permeation of OLP was significantly improved and supported the hypothesis that water-soluble polymers, such as PEG 3400, enhance the formation and stability of inclusion complexes.^49^

With regard to the *in vitro* permeation of OLP from SD DCTs using PVA/PVP MN arrays, further improvement in performance was recorded. After 24 h, 375.10 ± 68.02 μg (54.13 ± 9.81%) of OLP had permeated across dermatomed neonatal porcine skin, which was significantly greater than the permeation from FX DCTs using the same type of MN array (*p* = 0.0104). The drug permeation observed here was also significantly greater than the permeation observed when SD and PEG.SD DCTs were combined with Gantrez MN arrays, with *p* values of 0.0121 and 0.0243 recorded, respectively. This observation indicated that HP-β-CD did not interact with components of the PVA/PVP hydrogel to the same extent as the Gantrez hydrogel resulting in reduced dissociation of drug/CD inclusion complexes and improved transdermal permeation of OLP. This hypothesis is further bolstered by the fact that PVA/PVP arrays were less yellow in color following experimentation, and MN tips remained intact. Further increment in the permeation of OLP across porcine skin using hydrogel-forming MN arrays was achieved using PEG.SD DCTs. With these reservoirs, cumulative OLP permeation was 473.27 ± 40.44 μg (68.29 ± 5.84%), although statistical analysis revealed that this permeation was not significantly greater than that of SD DCTs (*p* = 0.1138). Nevertheless, when compared to the delivery of OLP from the poorest performing combination, which was composed of an FCD DCT paired with a Gantrez MN array (percentage permeation = 3.94 ± 1.24%), a 17-fold enhancement in the transdermal delivery of OLP was achieved. As a result, hydrogel-forming MAPs composed of PEG.SD DCTs and PVA/PVP MN arrays were taken forward for *in vivo* investigation.

### 3.2 Dissolving MAPs

#### 3.2.1 Unprocessed OLP Particle Size Determination

**Figure 8:**
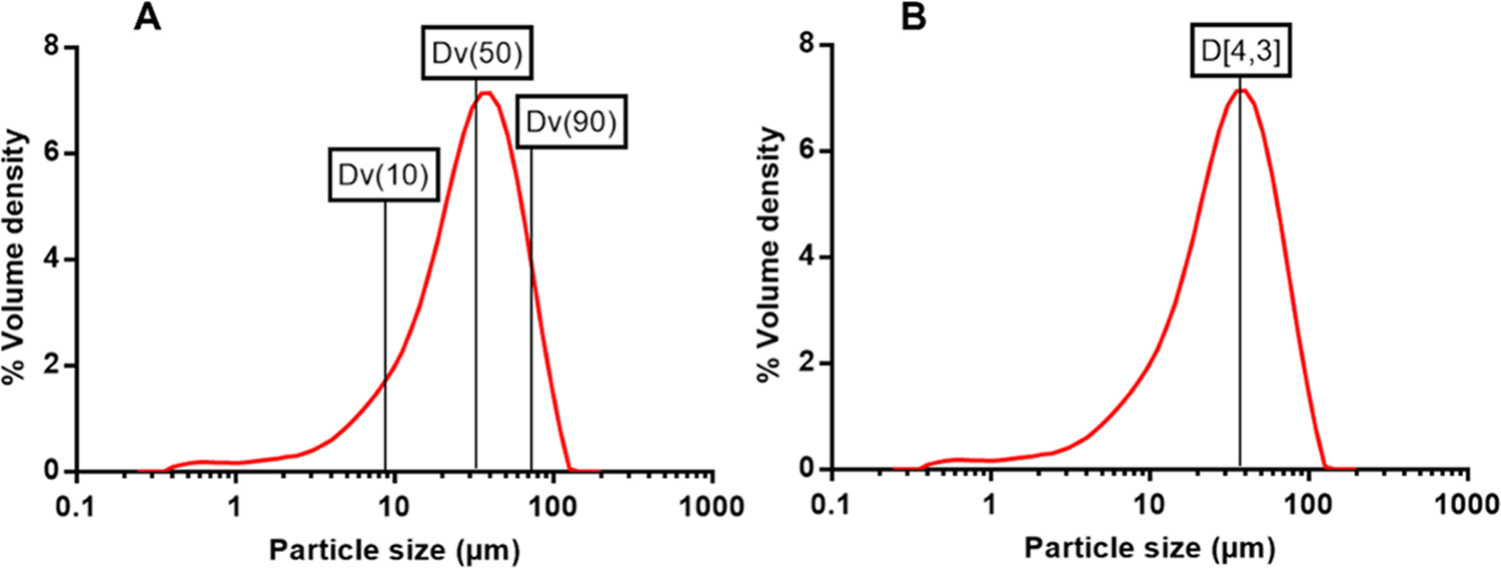
Particle size distribution of unprocessed olanzapine powder following analysis using the Malvern Mastersizer 3000, with (A) Dv(10), Dv(50), and Dv(90) values added, and (B) D[4.3] value added.

The Dv(10), Dv(50), Dv(90), and D[4,3] values of unprocessed OLP were 8.6, 32.9, 72.9, and 37.4 μm, respectively (Figure 8A,B). As the D[4,3] value is often considered to be a more accurate measurement of the average particle size of a sample containing large particles, this was considered to be the average particle size of unprocessed OLP powder.

#### 3.2.2 Rationalization of Surfactant Selection

**Figure 9:**
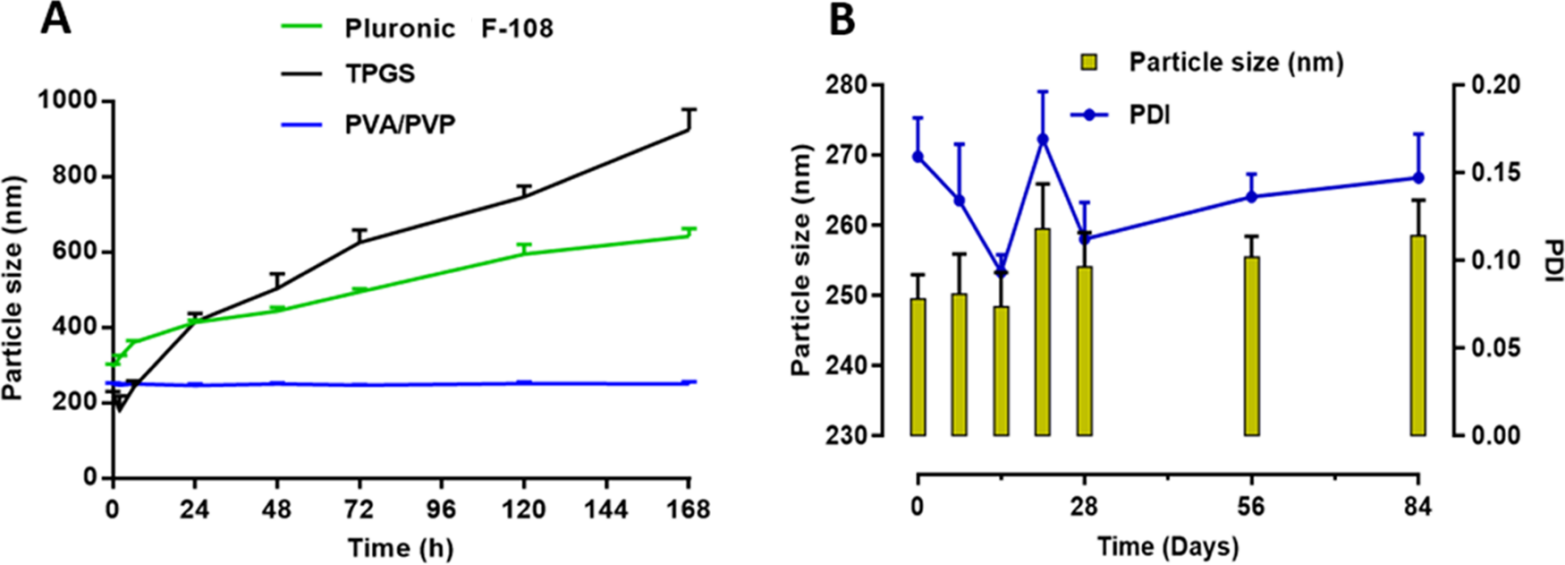
(A) Particle size of olanzapine nanocrystals in the presence of PVA/PVP (2% w/w) over the course of 1 week and (B) particle size and polydispersity index of olanzapine nanocrystals in the presence of PVA/PVP (2% w/w) over the course of 3 months (mean + S.D., *n* = 3).

When TPGS was used as the surfactant solution during milling, the OLP NCs produced had particle sizes of 224.27 ± 5.73 nm with a PDI of 0.144 ± 0.019. This was the lowest particle size and narrowest PDI observed during experimentation. These values rose somewhat to a particle size of 249.44 ± 3.49 nm and a PDI of 0.159 ± 0.022 when PVA/PVP was the chosen surfactant. The largest particle size and PDI, which were 289.66 ± 7.69 nm and 0.262 ± 0.087, respectively, were observed in samples where Pluronic F-108 was used as the surfactant. These early analyses indicated that TPGS was the most effective surfactant, when compared to PVA/PVP and Pluronic F-108. However, continued assessment of these NCs highlighted their propensity to aggregate, resulting in an increased particle size of 924.30 ± 54.43 nm at *t* = 168 h (Figure 9A). A similar particle size increase was observed in OLP NCs where Pluronic F-108 was the surfactant of choice, with particles measuring 641.81 ± 20.01 nm after 1 week. The phenomenon by which nanoparticles, such as NCs, spontaneously transition from small particles to larger particles within a formulation is termed “Ostwald ripening”.^50^ First described by Wilhelm Ostwald in 1896, this ripening or maturation effect occurs when small NCs dissolve within a formulation but, due to thermodynamic instability, they rapidly precipitate out of solution by depositing on the surface of larger particles.^50^ Over the course of this week-long particle size stability study, OLP NCs that had TPGS or Pluronic F-108 as the functional surface surfactant exhibited crystal growth consistent with Ostwald ripening and, therefore, were not selected for further investigation. Alternatively, OLP NCs formed in the presence of PVA/PVP demonstrated inherent stability in terms of particle size, with values remaining within the range of 246.40–251.03 nm throughout. Additionally, the PDI of NCs present remained below 0.2 in all cases. Following completion of the week-long particle size stability study, the stability of OLP NCs formed by wet bead milling in the presence of a PVA/PVP was investigated over 3 months (Figure 9B). The range of particle sizes measured was 246.40–259.47 nm (PDI remained below 0.2 in all cases), with no significant difference between OLP NCs at *t* = 0 and 84 days in terms of particles size and PDI (*p* > 0.05) observed. These findings further reinforced the perceived stability of OLP NCs when formulated with PVA/PVP as the surfactant of choice.

#### 3.2.3 Lyophilization of OLP NS to Produce OLP NC Powder

Aggregation of nanoparticles during the freeze-drying process is well documented in the literature, although the exact mechanisms of lyophilization-induced aggregation are not well understood.^51^ The particle isolation hypothesis suggests that in the absence of sufficient shielding from formulation excipients, nanoparticles interact with one another upon the removal of water, resulting in particulate growth.^51^ In this instance, PVA and PVP were used as shielding excipients, termed cryoprotectants, to interact with the surface of individual NCs and prevent interactions between particles. It is evident from Table 9 that the concentrations of each polymer were not sufficient enough to protect individual OLP NCs from aggregation upon lyophilization as a significant increase in both values had occurred following lyophilization of NS1. In subsequent samples, where the concentration of PVA and PVP was increased, smaller particle sizes and PDIs were observed. Only OLP NCs obtained following lyophilization of NS5 (OLP NS + 1 mL PVA/PVP 2% w/w solution) possessed a PDI below the acceptable limit of <0.2. Although aggregation still occurred within this formulation upon lyophilization, this finding confirmed that only NS5 possessed concentrations of PVA and PVP sufficient enough to provide adequate cryoprotection that ensured maintenance of acceptable particle size and PDI values. Consequently, the powder formulation prepared from freeze-dried NS5 was taken forward as a candidate formulation for the preparation of dissolving MAPs containing OLP NCs.

**Table 9:** Particle Size and Polydispersity Index of Olanzapine Nanocrystals Present within Nanosuspensions Containing Differing Concentrations of PVA/PVP 2% w/w Solution (Mean + or ± S.D., *n* = 3)

formulation	particle size (nm)	polydispersity index
NS1	1794.73 ± 76.50	0.543 ± 0.121
NS2	1208.91 ± 46.14	0.321 ± 0.087
NS3	502.60 ± 23.67	0.267 ± 0.061
NS4	391.07 ± 12.77	0.212 ± 0.043
NS5	340.82 ± 5.60	0.189 ± 0.027

#### 3.2.4 Characterization of Coarse OLP Powder and OLP NC Powder

##### 3.2.4.1 DSC, PXRD, SEM

**Figure 10:**
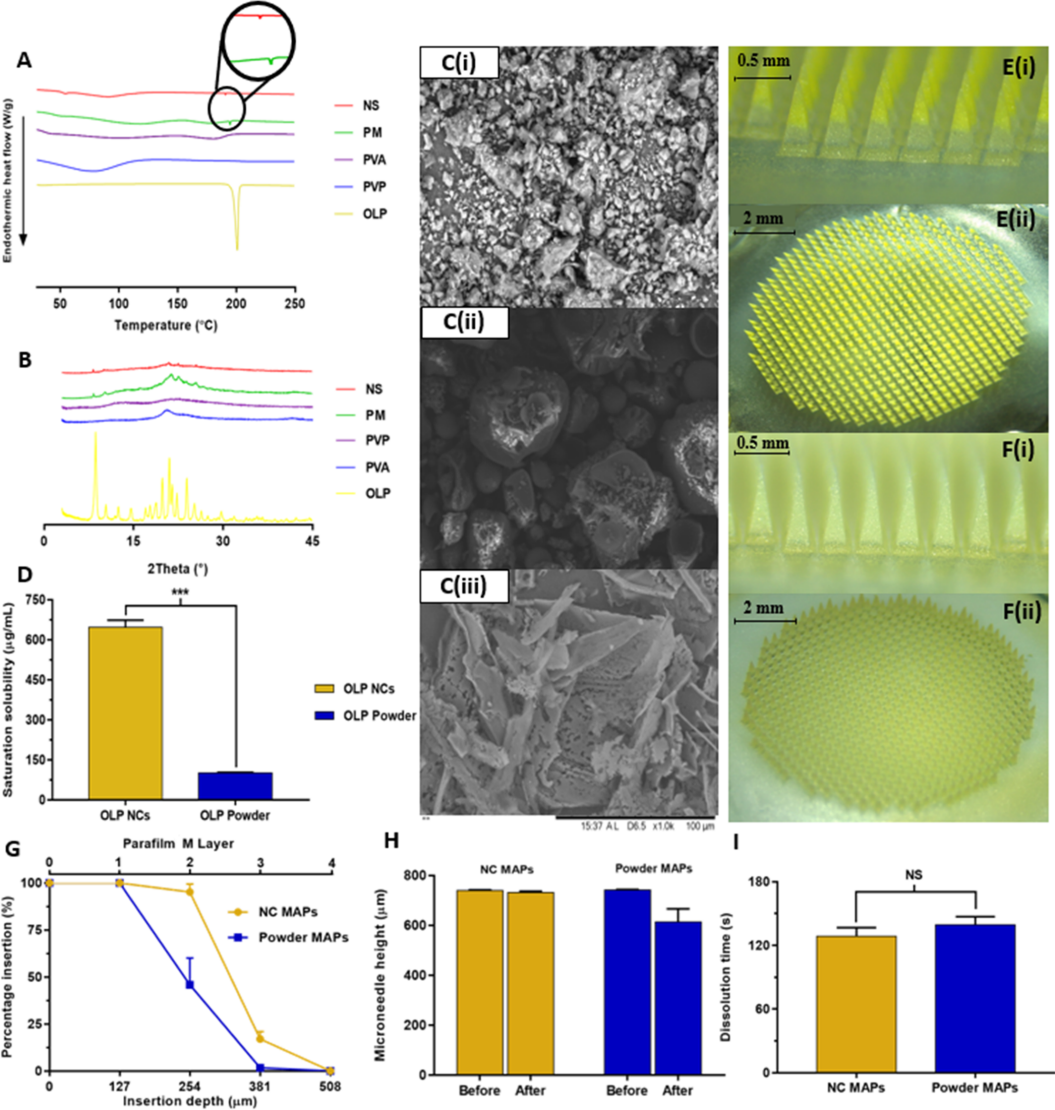
Traces obtained from the analysis of olanzapine, poly(vinyl alcohol), poly(vinyl pyrrolidone), a physical mixture of these components, and lyophilized olanzapine nanocrystal powder using (A) differential scanning calorimetry and (B) powder X-ray diffraction. Additionally, (C) SEM images obtained from the examination of (i) olanzapine, (ii) physical mixture, and (iii) lyophilized olanzapine nanocrystals at a magnification of ×1000, and (D) saturation solubilities of unprocessed olanzapine powder and olanzapine nanocrystals in PBS (pH 7.4). Representative light microscope images of dissolving microarray patches containing (E) unprocessed olanzapine powder at (i) ×35 magnification and (ii) ×8 magnification, and (F) olanzapine nanocrystals at (i) ×35 magnification and (ii) ×8 magnification. (G) Insertion efficiency of microneedles into an artificial skin model, (H) height of formulated microneedles pre- and post-insertion, and (I) dissolution time of formulated microneedles when placed in prewarmed PBS (37 ± 1 °C) (mean + S.D., *n* = 3, *** *p* < 0.001).

The sharp melting point peak observed on the DSC trace of OLP at ∼198 °C, as discussed previously, corresponds to the breakdown of its crystalline structure^44^ (Figure 10A). Similar endothermic peaks were observed on the trace of a physical mixture composed of OLP, PVA, and PVP, and the trace of OLP NS, although the intensity of each was greatly reduced. In a similar manner, the crystalline peaks observed on the PXRD diffractogram of OLP, at ∼8.62° and between 14.58–27.42°, were also observed on the diffractograms of PM and OLP NS (Figure 10B). These findings indicated that the crystalline structure of OLP had not been converted to one that was amorphous by the processes involved in NC formation.^24,52^ In the SEM image of pure OLP, small and irregularly shaped crystals were observed due to the drug’s inherent crystalline structure (Figure 10C(i)). When a PM of OLP and the polymers PVA and PVP were analyzed, larger particles with more rounded structures were observed in addition to the small crystals of OLP. Due to the processes of dissolution and lyophilization during NC powder preparation, the appearance of particles in Figure 10C(iii) was much less regular than that in Figure 10C(ii). In the absence of particles with similar surface morphologies to that of unprocessed OLP, which were present in Figure 10C(i and ii), it was assumed that individual OLP particles were not visible due to their size reduction to within the nanometer range.

The saturation solubility of OLP NCs in PBS was 646.45 ± 26.72 μg/mL, which was an approximately 6.5-fold enhancement compared to the intrinsic solubility of OLP. Improvement in the aqueous solubility of poorly soluble compounds when formulated into NCs is due to the increased overall surface area attained through particle size reduction.^2^

#### 3.2.5 Characterization of Dissolving MAPs

Although both formulations produced MNs with similar dimensions, the appearance of each MN type was distinctly different. In powder-containing MNs, OLP exists as large, micron-sized particles that exhibit poor solubility. Upon casting of this formulation onto MN moulds and subsequent exposure to positive pressure to fill MN cavities, these large, insoluble OLP particles sediment in the bottom of each MN cavity, resulting in the production of MNs with intensely colored yellow tips following drying (Figure 10E(i and ii)). In contrast, MNs containing OLP NCs have a homogeneous appearance due to the presence of much smaller OLP particles that exhibit increased solubility compared to unprocessed OLP powder and, therefore, no sedimentation during MAP preparation (Figure 10F(i and ii)).

Considering the insertion efficiency of the formulated MAPs, it can be seen in Figure 10G that all MNs on both patches were successfully inserted through the first layer of Parafilm M. In the second layer, which equated to an insertion depth of ∼254 μm, 95.22 ± 4.23% of MNs containing OLP NCs had inserted, whereas a significantly lower insertion efficiency value of 45.89 ± 14.24% for powder-containing MNs was observed (*p* = 0.0004). A similar result was observed for layer three, where the percentage insertions of NC and Powder MNs were 17.00 ± 3.96 and 1.75 ± 1.72%, respectively (*p* = 0.0007). Insertion through Parafilm M layer four (insertion depth ∼ 508 μm) was not observed for either MAP formulation. The superior insertion of NC-containing MAPs could be attributed to the previously described homogeneity of these MNs in terms of drug and polymer content. As a result, MN tips possessed sufficient strength and so were capable of inserting to greater depths without suffering mechanical failure. This hypothesis was supported by comparison of MN heights before and after insertion into the Parafilm M skin model where the height reduction of NC-containing MNs was significantly smaller than that of powder-containing MNs (*p* = 0.0011) (Figure 10H). While these findings indicated that OLP powder MAPs possessed MNs that were not strong enough to resist mechanical failure upon application, the potential of a MAP where a large proportion of drug payload is located in the very tip of MNs is considerable. No significant difference between MAP formulations in terms of dissolution time was recorded (*p* = 0.0625) with all NC-containing MNs dissolving within 128 ± 8.17 s and all powder-containing MNs dissolving within 139.40 ± 7.60 s (Figure 10I). This data confirmed that the presence of OLP, whether it is unprocessed or in NC form, does not prevent the dissolution of dissolving MNs. Subsequent analysis of dissolved MAPs revealed that drug loading of NC- and powder-containing MAPs was 1.34 ± 0.121 and 1.31 ± 0.125 mg, respectively. Additionally, the particle size and PDI of OLP NCs following MAP dissolution were 347.04 ± 3.40 nm and 0.141 ± 0.019, respectively. This finding confirmed that MAP formulation did not cause the particle size of OLP NCs to grow significantly (*p* = 0.1904).

#### 3.2.6 *In Vitro* Delivery of OLP from Dissolving MAPs

**Figure 11:**
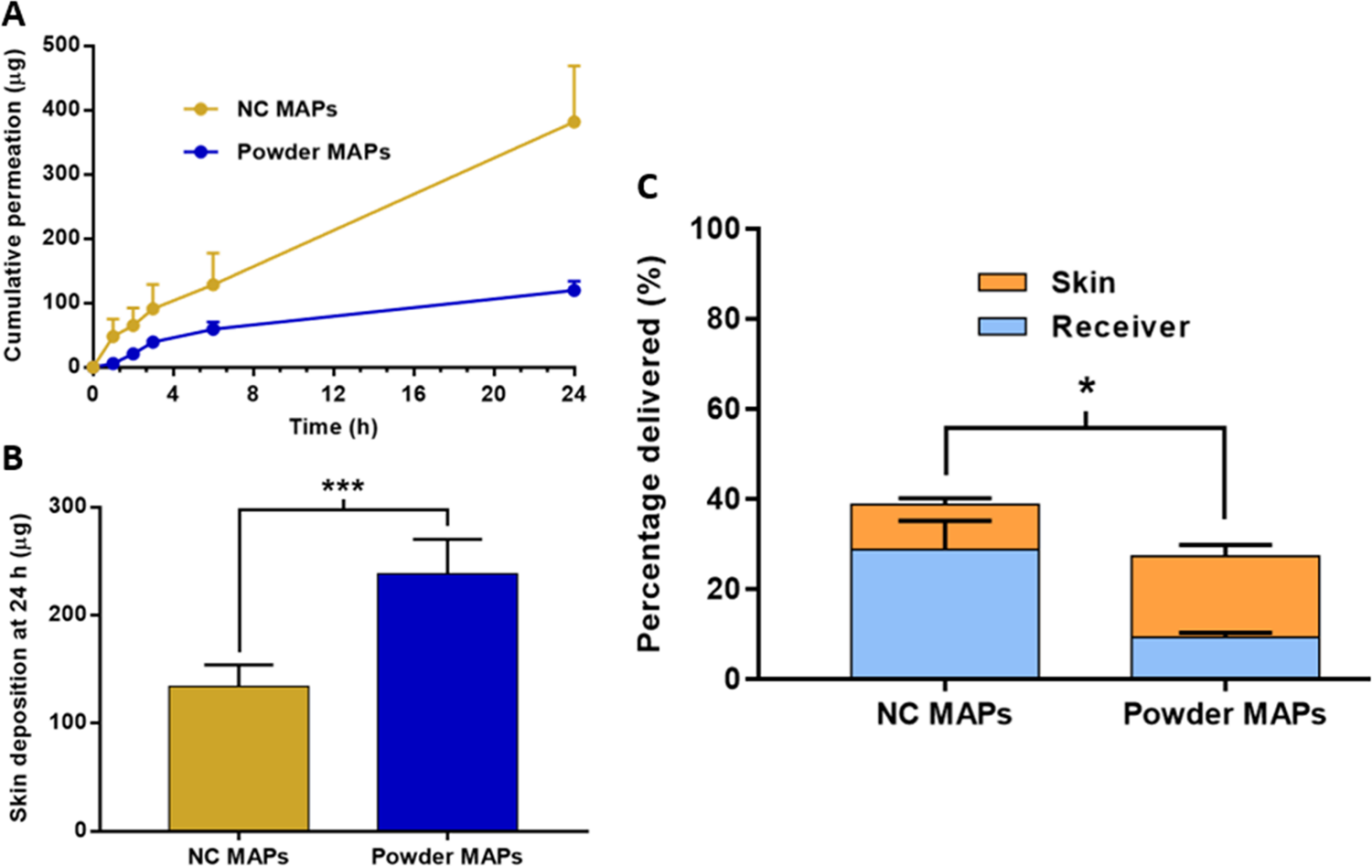
Transdermal delivery of olanzapine from microarray patches containing either olanzapine nanocrystals or unprocessed olanzapine powder. (A) Cumulative permeation across full-thickness porcine skin over 24 h, (B) amount of olanzapine deposited in full-thickness neonatal porcine skin after 24 h, and (C) overall delivery profiles after 24 h (mean + S.D., *n* = 5, * 0.05 > *p* > 0.01).

Over the course of 24 h, the cumulative transdermal permeation values, *i.e.*, across porcine skin and into the Franz cell receiver compartment, of OLP from NC MAPs and powder MAPs were 382.01 ± 87.36 and 119.76 ± 14.39 μg, respectively (Figure 11A). The significantly greater transdermal permeation of OLP from NC MAPs (*p* = 0.0022) was attributed to the increased solubility of OLP NCs compared to unprocessed OLP powder. The epidermis and dermis layers of the skin are hydrophilic environments and so drug particles with enhanced aqueous solubility are able to permeate across, and through, these layers with greater ease than less soluble drug particles. In contrast, the results for intradermal drug deposition indicated that MAPs containing OLP in its unprocessed powder form were superior to NC-containing MAPs (*p* = 0.0006) with each depositing 238.13 ± 32.25 and 133.79 ± 20.30 μg, respectively (Figure 11B).

Once again, this result was attributed to the difference in aqueous solubility between unprocessed OLP powder and OLP NCs. Upon dissolution of MNs containing less soluble OLP powder, a lower proportion of the drug payload is solubilized within the hydrophilic environment of the skin; instead, much of the drug remains out of solution and crystallized within the skin. A recent publication from Tekko et al. reported a similar finding where considerable levels of drug deposited within the skin following delivery of the poorly soluble HIV therapeutic cabotegravir *via* comparably formulated dissolving MAPs.^53^ Considering the overall delivery profile from the formulated MAPs (Figure 11C), those containing OLP NCs delivered 38.61 ± 8.06% after 24 h, while those containing unprocessed OLP delivered 27.24 ± 3.55%, which was significantly less (*p* = 0.0307). For NC-containing MAPs, more OLP was delivered across the skin (28.59 ± 6.54%) than was deposited in the skin (10.01 ± 1.52%). However, with MAPs containing OLP powder, more drug was deposited in the skin (18.12 ± 2.45%) than was delivered across the skin (9.11 ± 1.10%). The differences observed between these delivery profiles in terms of drug distribution following MAP administration illustrated the effect that particle size can have on the percutaneous delivery of poorly soluble therapeutics. Based on the superior performance of OLP NC MAPs, in terms of insertion capability, mechanical strength, and overall drug delivery in an *in vitro* setting, this formulation was selected as the most suitable candidate to be taken forward for *in vivo* testing.

### 3.3 *In Vivo* Delivery of OLP from Dissolving and Hydrogel-Forming MAPs

**Figure 12:**
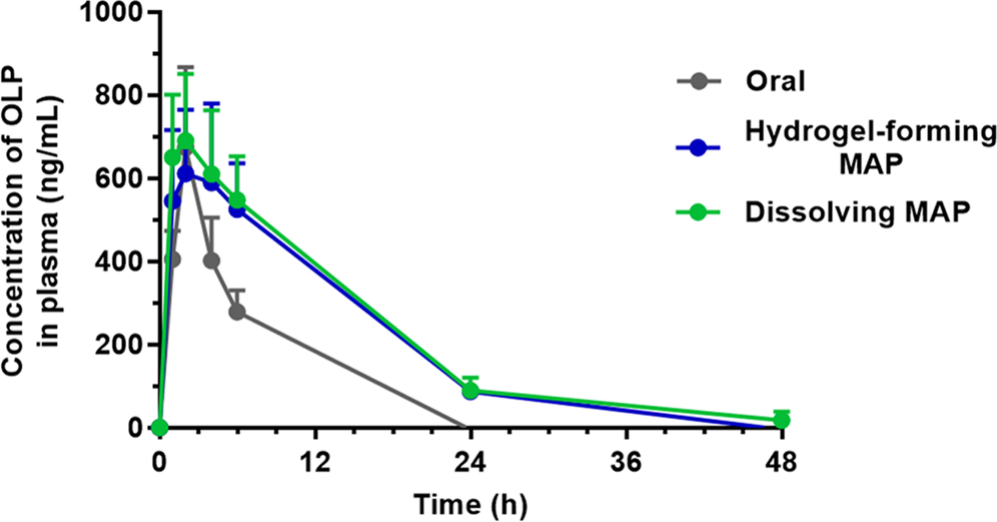
*In vivo* delivery profiles of olanzapine delivered *via* either the oral route using an oral suspension or the transdermal route using nanocrystal-containing dissolving microarray patches or spray-dried cyclodextrin complex-containing hydrogel-forming microarray patches (mean + S.D., *n* = 6).

The maximum plasma concentration of OLP when delivered *via* NC-containing dissolving MAPs (690.56 ± 161.33 ng/mL) was observed at 2 h post-MAP application (Figure 12). This value was statistically similar to that of orally administered OLP where a *C*_max_ of 673.36 ± 193.50 ng/mL was observed at the same timepoint (*p* = 0.8706). At 24 h post-administration, OLP was not detected in any of the animals that were treated orally. In contrast, the plasma concentration of OLP in rats that were treated with dissolving MAPs was 89.20 ± 30.81 ng/mL at the same timepoint and 17.74 ± 11.55 ng/mL at *t* = 48 h. Regarding overall OLP exposure, the AUC values of the delivery profiles of OLP administered *via* oral gavage and dissolving MAPs were 5487 ± 532.90 and 10470 ± 1133.07, respectively. Additionally, based on an oral bioavailability value of 57% (obtained from the literature as detailed previously), the bioavailability of OLP delivered *via* dissolving MAPs was calculated to be 59% (Table 10).

**Table 10:** Pharmacokinetic Parameters of the Delivery of Olanzapine *via* Oral Suspension, Dissolving Microarray Patch, and Hydrogel-Forming Microarray Patch (Mean ± S.D)

pharmacokinetic parameter	oral suspension	dissolving MAP	hydrogel-forming MAP
*C*_max_ (ng/mL)	673.36 ± 193.50	690.56 ± 161.33	611.13 ± 153.34
*T*_max_ (h)	2	2	2
AUC	5487 ± 532.90	10470 ± 1133.07	9670 ± 1076.10
bioavailability (%)	57^a^	59	54

a Value obtained from the literature.

When delivered *via* CD complex-containing hydrogel-forming MAPs, the maximum plasma concentration of OLP (611.13 ± 153.34 ng/mL) was also observed at 2 h post-MAP application. These values were statistically similar to that of OLP administered both orally and *via* NC-containing dissolving MAPs (*p* = 0.7026). At 24 h post-administration, the plasma concentration of OLP in rats in this cohort was 86.90 ± 10.64 ng/mL, which was statistically similar to that observed in rats treated with dissolving MAPs (*p* = 0.8680). As mentioned previously, OLP was not detected in the oral cohort at the 24 h timepoint. Regarding the overall OLP exposure, the AUC of the delivery profile of OLP administered *via* hydrogel-forming MAPs was 9670 ± 1076.10. This was statistically similar to that of NC-containing dissolving MAPs (*p* = 0.2384) and significantly greater than the AUC of the oral delivery profile (*p* < 0.0001). Finally, based on an oral bioavailability value of 57% (obtained from the literature), the bioavailability of OLP delivered *via* hydrogel-forming MAPs was calculated to be 54% (Table 10).

The similarities observed at early timepoints (0–2 h) between the delivery profiles of OLP from both MAP types and an oral suspension of OLP indicated the initial rate and extent of OLP absorption following administration was similar. This finding supports the hypothesis that, although MAPs have primarily been utilized for the delivery of hydrophilic compounds, hydrophobic compounds can also be effectively delivered using this platform when combined with suitable solubility enhancement strategies. Regarding the extended exposure to OLP of rats treated with MAPs, the AUC values obtained for each delivery profile suggested that this may have been a dose-dependent observation, *i.e.*, the drug loading and the AUC of each MAP type was twice that of the orally administered suspension. However, as OLP was not detected at the 24 h timepoint, the AUC obtained for oral OLP may have been an overestimate due to the absence of sampling timepoints between 6 and 24 h. The potential for overestimation regarding the AUC of the oral delivery profile suggests that the extended release of OLP from MAPs may be influenced by other factors, as well as the administered dose. One factor that may be influential is continued and controlled permeation of OLP into, and through, the skin during the 24 h MAP application period.^12^ This is of particular importance with hydrogel-forming MAPs where continuous intradermal infusion of drugs can be observed for as long as patches are applied.^54^ Throughout this period, the opportunity for transdermal OLP permeation was ample, whereas OLP absorption following oral administration may have been adversely affected by environmental factors in the stomach, such as gastric emptying.

The findings obtained from the comparison of these delivery profiles confirmed that the formulated MAPs were capable of delivering OLP in a manner that was at least similar to that of oral administration. As this work was concept-proving in nature, future work could focus on the refinement and optimization of each MAP formulation to produce a delivery profile that is even more sustained. For dissolving MAPs, alterations in the drug content of individual MNs and increasing overall patch size would greatly influence drug loading. Of course, maintaining the structural integrity of MNs and ensuring successful application of MAPs with larger surface areas would be of paramount importance. Crucial to the success of the dissolving MAP platform, not just for the delivery of OLP but as a whole, is complete and thorough characterization of repeated MAP application and polymer deposition in the skin. For hydrogel-forming MAPs, variation in polymer composition and cross-link density will directly influence intradermal drug permeation from a hydrogel patch. Furthermore, extending patch wear time and maximizing reservoir drug loading may also serve to extend drug release profiles without having to increase the patch size.

## 4 Conclusions

There is an ever-increasing trend in the number of new chemical entities and lead drug candidates that exhibit an inherent lack of adequate aqueous solubility. As a result, there is an unmet need for new and alternative drug delivery strategies that can successfully deliver such therapeutics. This work sought to demonstrate how polymeric MAPs, when combined with effective solubility enhancement strategies, can expand the library of molecules amenable to transdermal delivery to include those that are poorly soluble. To achieve this, the solubility of OLP was enhanced *via* either a novel CD complexation strategy or particle size reduction, before robust OLP-containing hydrogel-forming and dissolving MAPs were formulated and characterized *in vitro*. When tested *in vivo*, successful delivery of OLP, a poorly soluble drug that is unsuited to transdermal permeation, *via* the formulated MAPs was achieved with similar onset and an extended exposure compared to oral administration. Future work will focus on the modulation of both hydrogel-forming and dissolving MAP formulations to further extend drug delivery following application and, crucially, investigation of the key performance parameters of polymeric MAPs as a platform to accelerate their translation to the market.
